# Measurement of electroweak production of a $\mathrm{W}$ boson in association with two jets in proton–proton collisions at $\sqrt{s}=13\,\text {Te}\text {V}$

**DOI:** 10.1140/epjc/s10052-019-7585-7

**Published:** 2020-01-18

**Authors:** A. M. Sirunyan, A. Tumasyan, W. Adam, F. Ambrogi, E. Asilar, T. Bergauer, J. Brandstetter, M. Dragicevic, J. Erö, A. Escalante Del Valle, M. Flechl, R. Frühwirth, V. M. Ghete, J. Hrubec, M. Jeitler, N. Krammer, I. Krätschmer, D. Liko, T. Madlener, I. Mikulec, N. Rad, H. Rohringer, J. Schieck, R. Schöfbeck, M. Spanring, D. Spitzbart, W. Waltenberger, J. Wittmann, C.-E. Wulz, M. Zarucki, V. Chekhovsky, V. Mossolov, J. Suarez Gonzalez, E. A. De Wolf, D. Di Croce, X. Janssen, J. Lauwers, A. Lelek, M. Pieters, H. Van Haevermaet, P. Van Mechelen, N. Van Remortel, F. Blekman, J. D’Hondt, J. De Clercq, K. Deroover, G. Flouris, D. Lontkovskyi, S. Lowette, I. Marchesini, S. Moortgat, L. Moreels, Q. Python, K. Skovpen, S. Tavernier, W. Van Doninck, P. Van Mulders, I. Van Parijs, D. Beghin, B. Bilin, H. Brun, B. Clerbaux, G. De Lentdecker, H. Delannoy, B. Dorney, L. Favart, A. Grebenyuk, A. K. Kalsi, J. Luetic, A. Popov, N. Postiau, E. Starling, L. Thomas, C. Vander Velde, P. Vanlaer, D. Vannerom, Q. Wang, T. Cornelis, D. Dobur, A. Fagot, M. Gul, I. Khvastunov, C. Roskas, D. Trocino, M. Tytgat, W. Verbeke, B. Vermassen, M. Vit, N. Zaganidis, O. Bondu, G. Bruno, C. Caputo, P. David, C. Delaere, M. Delcourt, A. Giammanco, G. Krintiras, V. Lemaitre, A. Magitteri, K. Piotrzkowski, A. Saggio, M. Vidal Marono, P. Vischia, J. Zobec, F. L. Alves, G. A. Alves, G. Correia Silva, C. Hensel, A. Moraes, M. E. Pol, P. Rebello Teles, E. Belchior Batista Das Chagas, W. Carvalho, J. Chinellato, E. Coelho, E. M. Da Costa, G. G. Da Silveira, D. De Jesus Damiao, C. De Oliveira Martins, S. Fonseca De Souza, L. M. Huertas Guativa, H. Malbouisson, D. Matos Figueiredo, M. Melo De Almeida, C. Mora Herrera, L. Mundim, H. Nogima, W. L. Prado Da Silva, L. J. Sanchez Rosas, A. Santoro, A. Sznajder, M. Thiel, E. J. Tonelli Manganote, F. Torres Da Silva De Araujo, A. Vilela Pereira, S. Ahuja, C. A. Bernardes, L. Calligaris, T. R. Fernandez Perez Tomei, E. M. Gregores, P. G. Mercadante, S. F. Novaes, SandraS. Padula, A. Aleksandrov, R. Hadjiiska, P. Iaydjiev, A. Marinov, M. Misheva, M. Rodozov, M. Shopova, G. Sultanov, A. Dimitrov, L. Litov, B. Pavlov, P. Petkov, W. Fang, X. Gao, L. Yuan, M. Ahmad, J. G. Bian, G. M. Chen, H. S. Chen, M. Chen, Y. Chen, C. H. Jiang, D. Leggat, H. Liao, Z. Liu, S. M. Shaheen, A. Spiezia, J. Tao, E. Yazgan, H. Zhang, S. Zhang, J. Zhao, Y. Ban, G. Chen, A. Levin, J. Li, L. Li, Q. Li, Y. Mao, S. J. Qian, D. Wang, Y. Wang, C. Avila, A. Cabrera, C. A. Carrillo Montoya, L. F. Chaparro Sierra, C. Florez, C. F. González Hernández, M. A. Segura Delgado, J. D. Ruiz Alvarez, N. Godinovic, D. Lelas, I. Puljak, T. Sculac, Z. Antunovic, M. Kovac, V. Brigljevic, D. Ferencek, K. Kadija, B. Mesic, M. Roguljic, A. Starodumov, T. Susa, M. W. Ather, A. Attikis, M. Kolosova, S. Konstantinou, G. Mavromanolakis, J. Mousa, C. Nicolaou, F. Ptochos, P. A. Razis, H. Rykaczewski, M. Finger, M. Finger, E. Ayala, E. Carrera Jarrin, A. A. Abdelalim, A. Ellithi Kamel, E. Salama, S. Bhowmik, A. Carvalho Antunes De Oliveira, R. K. Dewanjee, K. Ehataht, M. Kadastik, M. Raidal, C. Veelken, P. Eerola, H. Kirschenmann, J. Pekkanen, M. Voutilainen, J. Havukainen, J. K. Heikkilä, T. Järvinen, V. Karimäki, R. Kinnunen, T. Lampén, K. Lassila-Perini, S. Laurila, S. Lehti, T. Lindén, P. Luukka, T. Mäenpää, H. Siikonen, E. Tuominen, J. Tuominiemi, T. Tuuva, M. Besancon, F. Couderc, M. Dejardin, D. Denegri, J. L. Faure, F. Ferri, S. Ganjour, A. Givernaud, P. Gras, G. Hamel de Monchenault, P. Jarry, C. Leloup, E. Locci, J. Malcles, J. Rander, A. Rosowsky, M. Ö. Sahin, A. Savoy-Navarro, M. Titov, C. Amendola, F. Beaudette, P. Busson, C. Charlot, B. Diab, R. Granier de Cassagnac, I. Kucher, A. Lobanov, J. Martin Blanco, C. Martin Perez, M. Nguyen, C. Ochando, G. Ortona, P. Paganini, J. Rembser, R. Salerno, J. B. Sauvan, Y. Sirois, A. Zabi, A. Zghiche, J.-L. Agram, J. Andrea, D. Bloch, G. Bourgatte, J.-M. Brom, E. C. Chabert, C. Collard, E. Conte, J.-C. Fontaine, D. Gelé, U. Goerlach, M. Jansová, A.-C. Le Bihan, N. Tonon, P. Van Hove, S. Gadrat, S. Beauceron, C. Bernet, G. Boudoul, N. Chanon, R. Chierici, D. Contardo, P. Depasse, H. El Mamouni, J. Fay, S. Gascon, M. Gouzevitch, G. Grenier, B. Ille, F. Lagarde, I. B. Laktineh, H. Lattaud, M. Lethuillier, L. Mirabito, S. Perries, V. Sordini, G. Touquet, M. Vander Donckt, S. Viret, T. Toriashvili, I. Bagaturia, C. Autermann, L. Feld, M. K. Kiesel, K. Klein, M. Lipinski, D. Meuser, A. Pauls, M. Preuten, M. P. Rauch, C. Schomakers, J. Schulz, M. Teroerde, B. Wittmer, A. Albert, M. Erdmann, S. Erdweg, T. Esch, R. Fischer, S. Ghosh, T. Hebbeker, C. Heidemann, K. Hoepfner, H. Keller, L. Mastrolorenzo, M. Merschmeyer, A. Meyer, P. Millet, S. Mukherjee, A. Novak, T. Pook, A. Pozdnyakov, M. Radziej, H. Reithler, M. Rieger, A. Schmidt, A. Sharma, D. Teyssier, S. Thüer, G. Flügge, O. Hlushchenko, T. Kress, T. Müller, A. Nehrkorn, A. Nowack, C. Pistone, O. Pooth, D. Roy, H. Sert, A. Stahl, M. Aldaya Martin, T. Arndt, C. Asawatangtrakuldee, I. Babounikau, H. Bakhshiansohi, K. Beernaert, O. Behnke, U. Behrens, A. Bermúdez Martínez, D. Bertsche, A. A. Bin Anuar, K. Borras, V. Botta, A. Campbell, P. Connor, C. Contreras-Campana, V. Danilov, A. De Wit, M. M. Defranchis, C. Diez Pardos, D. Domínguez Damiani, G. Eckerlin, T. Eichhorn, A. Elwood, E. Eren, E. Gallo, A. Geiser, J. M. Grados Luyando, A. Grohsjean, M. Guthoff, M. Haranko, A. Harb, N. Z. Jomhari, H. Jung, M. Kasemann, J. Keaveney, C. Kleinwort, J. Knolle, D. Krücker, W. Lange, T. Lenz, J. Leonard, K. Lipka, W. Lohmann, R. Mankel, I.-A. Melzer-Pellmann, A. B. Meyer, M. Meyer, M. Missiroli, G. Mittag, J. Mnich, V. Myronenko, S. K. Pflitsch, D. Pitzl, A. Raspereza, A. Saibel, M. Savitskyi, P. Saxena, V. Scheurer, P. Schütze, C. Schwanenberger, R. Shevchenko, A. Singh, H. Tholen, O. Turkot, A. Vagnerini, M. Van De Klundert, G. P. Van Onsem, R. Walsh, Y. Wen, K. Wichmann, C. Wissing, O. Zenaiev, R. Aggleton, S. Bein, L. Benato, A. Benecke, V. Blobel, T. Dreyer, A. Ebrahimi, E. Garutti, D. Gonzalez, P. Gunnellini, J. Haller, A. Hinzmann, A. Karavdina, G. Kasieczka, R. Klanner, R. Kogler, N. Kovalchuk, S. Kurz, V. Kutzner, J. Lange, D. Marconi, J. Multhaup, M. Niedziela, C. E. N. Niemeyer, D. Nowatschin, A. Perieanu, A. Reimers, O. Rieger, C. Scharf, P. Schleper, S. Schumann, J. Schwandt, J. Sonneveld, H. Stadie, G. Steinbrück, F. M. Stober, M. Stöver, B. Vormwald, I. Zoi, M. Akbiyik, C. Barth, M. Baselga, S. Baur, T. Berger, E. Butz, R. Caspart, T. Chwalek, W. De Boer, A. Dierlamm, K. El Morabit, N. Faltermann, M. Giffels, M. A. Harrendorf, F. Hartmann, U. Husemann, I. Katkov, S. Kudella, S. Mitra, M. U. Mozer, Th. Müller, M. Musich, G. Quast, K. Rabbertz, M. Schröder, I. Shvetsov, H. J. Simonis, R. Ulrich, M. Weber, C. Wöhrmann, R. Wolf, G. Anagnostou, G. Daskalakis, T. Geralis, A. Kyriakis, D. Loukas, G. Paspalaki, A. Agapitos, G. Karathanasis, P. Kontaxakis, A. Panagiotou, I. Papavergou, N. Saoulidou, K. Vellidis, G. Bakas, K. Kousouris, I. Papakrivopoulos, G. Tsipolitis, I. Evangelou, C. Foudas, P. Gianneios, P. Katsoulis, P. Kokkas, S. Mallios, K. Manitara, N. Manthos, I. Papadopoulos, E. Paradas, J. Strologas, F. A. Triantis, D. Tsitsonis, M. Bartók, M. Csanad, N. Filipovic, P. Major, K. Mandal, A. Mehta, M. I. Nagy, G. Pasztor, O. Surányi, G. I. Veres, G. Bencze, C. Hajdu, D. Horvath, Hunyadi, F. Sikler, T. Vámi, V. Veszpremi, G. Vesztergombi, N. Beni, S. Czellar, J. Karancsi, A. Makovec, J. Molnar, Z. Szillasi, P. Raics, Z. L. Trocsanyi, B. Ujvari, S. Choudhury, J. R. Komaragiri, P. C. Tiwari, S. Bahinipati, C. Kar, P. Mal, A. Nayak, S. Roy Chowdhury, D. K. Sahoo, S. K. Swain, S. Bansal, S. B. Beri, V. Bhatnagar, S. Chauhan, R. Chawla, N. Dhingra, R. Gupta, A. Kaur, M. Kaur, S. Kaur, P. Kumari, M. Lohan, M. Meena, K. Sandeep, S. Sharma, J. B. Singh, A. K. Virdi, G. Walia, A. Bhardwaj, B. C. Choudhary, R. B. Garg, M. Gola, S. Keshri, Ashok Kumar, S. Malhotra, M. Naimuddin, P. Priyanka, K. Ranjan, Aashaq Shah, R. Sharma, R. Bhardwaj, M. Bharti, R. Bhattacharya, S. Bhattacharya, U. Bhawandeep, D. Bhowmik, S. Dey, S. Dutt, S. Dutta, S. Ghosh, M. Maity, K. Mondal, S. Nandan, A. Purohit, P. K. Rout, A. Roy, G. Saha, S. Sarkar, T. Sarkar, M. Sharan, B. Singh, S. Thakur, P. K. Behera, A. Muhammad, R. Chudasama, D. Dutta, V. Jha, V. Kumar, D. K. Mishra, P. K. Netrakanti, L. M. Pant, P. Shukla, P. Suggisetti, T. Aziz, M. A. Bhat, S. Dugad, G. B. Mohanty, N. Sur, RavindraKumar Verma, S. Banerjee, S. Bhattacharya, S. Chatterjee, P. Das, M. Guchait, Sa. Jain, S. Karmakar, S. Kumar, G. Majumder, K. Mazumdar, N. Sahoo, S. Sawant, S. Chauhan, S. Dube, V. Hegde, A. Kapoor, K. Kothekar, S. Pandey, A. Rane, A. Rastogi, S. Sharma, S. Chenarani, E. Eskandari Tadavani, S. M. Etesami, M. Khakzad, M. Mohammadi Najafabadi, M. Naseri, F. Rezaei Hosseinabadi, B. Safarzadeh, M. Zeinali, M. Felcini, M. Grunewald, M. Abbrescia, C. Calabria, A. Colaleo, D. Creanza, L. Cristella, N. De Filippis, M. De Palma, A. Di Florio, F. Errico, L. Fiore, A. Gelmi, G. Iaselli, M. Ince, S. Lezki, G. Maggi, M. Maggi, G. Miniello, S. My, S. Nuzzo, A. Pompili, G. Pugliese, R. Radogna, A. Ranieri, G. Selvaggi, L. Silvestris, R. Venditti, P. Verwilligen, G. Abbiendi, C. Battilana, D. Bonacorsi, L. Borgonovi, S. Braibant-Giacomelli, R. Campanini, P. Capiluppi, A. Castro, F. R. Cavallo, S. S. Chhibra, G. Codispoti, M. Cuffiani, G. M. Dallavalle, F. Fabbri, A. Fanfani, E. Fontanesi, P. Giacomelli, C. Grandi, L. Guiducci, F. Iemmi, S. Lo Meo, S. Marcellini, G. Masetti, A. Montanari, F. L. Navarria, A. Perrotta, F. Primavera, A. M. Rossi, T. Rovelli, G. P. Siroli, N. Tosi, S. Albergo, A. Di Mattia, R. Potenza, A. Tricomi, C. Tuve, G. Barbagli, K. Chatterjee, V. Ciulli, C. Civinini, R. D’Alessandro, E. Focardi, G. Latino, P. Lenzi, M. Meschini, S. Paoletti, L. Russo, G. Sguazzoni, D. Strom, L. Viliani, L. Benussi, S. Bianco, F. Fabbri, D. Piccolo, F. Ferro, R. Mulargia, E. Robutti, S. Tosi, A. Benaglia, A. Beschi, F. Brivio, V. Ciriolo, S. Di Guida, M. E. Dinardo, S. Fiorendi, S. Gennai, A. Ghezzi, P. Govoni, M. Malberti, S. Malvezzi, D. Menasce, F. Monti, L. Moroni, M. Paganoni, D. Pedrini, S. Ragazzi, T. Tabarelli de Fatis, D. Zuolo, S. Buontempo, N. Cavallo, A. De Iorio, A. Di Crescenzo, F. Fabozzi, F. Fienga, G. Galati, A. O. M. Iorio, L. Lista, S. Meola, P. Paolucci, C. Sciacca, E. Voevodina, P. Azzi, N. Bacchetta, D. Bisello, A. Boletti, A. Bragagnolo, R. Carlin, P. Checchia, M. Dall’Osso, P. De Castro Manzano, T. Dorigo, U. Dosselli, F. Gasparini, U. Gasparini, A. Gozzelino, S. Y. Hoh, S. Lacaprara, P. Lujan, M. Margoni, A. T. Meneguzzo, J. Pazzini, M. Presilla, P. Ronchese, R. Rossin, F. Simonetto, A. Tiko, E. Torassa, M. Tosi, M. Zanetti, P. Zotto, G. Zumerle, A. Braghieri, A. Magnani, P. Montagna, S. P. Ratti, V. Re, M. Ressegotti, C. Riccardi, P. Salvini, I. Vai, P. Vitulo, M. Biasini, G. M. Bilei, C. Cecchi, D. Ciangottini, L. Fanò, P. Lariccia, R. Leonardi, E. Manoni, G. Mantovani, V. Mariani, M. Menichelli, A. Rossi, A. Santocchia, D. Spiga, K. Androsov, P. Azzurri, G. Bagliesi, L. Bianchini, T. Boccali, L. Borrello, R. Castaldi, M. A. Ciocci, R. Dell’Orso, G. Fedi, F. Fiori, L. Giannini, A. Giassi, M. T. Grippo, F. Ligabue, E. Manca, G. Mandorli, A. Messineo, F. Palla, A. Rizzi, G. Rolandi, A. Scribano, P. Spagnolo, R. Tenchini, G. Tonelli, A. Venturi, P. G. Verdini, L. Barone, F. Cavallari, M. Cipriani, D. Del Re, E. Di Marco, M. Diemoz, S. Gelli, E. Longo, B. Marzocchi, P. Meridiani, G. Organtini, F. Pandolfi, R. Paramatti, F. Preiato, C. Quaranta, S. Rahatlou, C. Rovelli, F. Santanastasio, N. Amapane, R. Arcidiacono, S. Argiro, M. Arneodo, N. Bartosik, R. Bellan, C. Biino, A. Cappati, N. Cartiglia, F. Cenna, S. Cometti, M. Costa, R. Covarelli, N. Demaria, B. Kiani, C. Mariotti, S. Maselli, E. Migliore, V. Monaco, E. Monteil, M. Monteno, M. M. Obertino, L. Pacher, N. Pastrone, M. Pelliccioni, G. L. Pinna Angioni, A. Romero, M. Ruspa, R. Sacchi, R. Salvatico, K. Shchelina, V. Sola, A. Solano, D. Soldi, A. Staiano, S. Belforte, V. Candelise, M. Casarsa, F. Cossutti, A. Da Rold, G. Della Ricca, F. Vazzoler, A. Zanetti, D. H. Kim, G. N. Kim, M. S. Kim, J. Lee, S. W. Lee, C. S. Moon, Y. D. Oh, S. I. Pak, S. Sekmen, D. C. Son, Y. C. Yang, H. Kim, D. H. Moon, G. Oh, B. Francois, J. Goh, T. J. Kim, S. Cho, S. Choi, Y. Go, D. Gyun, S. Ha, B. Hong, Y. Jo, K. Lee, K. S. Lee, S. Lee, J. Lim, S. K. Park, Y. Roh, H. S. Kim, J. Almond, J. Kim, J. S. Kim, H. Lee, K. Lee, S. Lee, K. Nam, S. B. Oh, B. C. Radburn-Smith, S. h. Seo, U. K. Yang, H. D. Yoo, G. B. Yu, D. Jeon, H. Kim, J. H. Kim, J. S. H. Lee, I. C. Park, Y. Choi, C. Hwang, J. Lee, I. Yu, V. Veckalns, V. Dudenas, A. Juodagalvis, J. Vaitkus, Z. A. Ibrahim, M. A. B. Md Ali, F. Mohamad Idris, W. A. T. Wan Abdullah, M. N. Yusli, Z. Zolkapli, J. F. Benitez, A. Castaneda Hernandez, J. A. Murillo Quijada, H. Castilla-Valdez, E. De La Cruz-Burelo, M. C. Duran-Osuna, I. Heredia-De La Cruz, R. Lopez-Fernandez, R. I. Rabadan-Trejo, G. Ramirez-Sanchez, R. Reyes-Almanza, A. Sanchez-Hernandez, S. Carrillo Moreno, C. Oropeza Barrera, M. Ramirez-Garcia, F. Vazquez Valencia, J. Eysermans, I. Pedraza, H. A. Salazar Ibarguen, C. Uribe Estrada, A. Morelos Pineda, N. Raicevic, D. Krofcheck, S. Bheesette, P. H. Butler, A. Ahmad, M. Ahmad, M. I. Asghar, Q. Hassan, H. R. Hoorani, W. A. Khan, M. A. Shah, M. Shoaib, M. Waqas, H. Bialkowska, M. Bluj, B. Boimska, T. Frueboes, M. Górski, M. Kazana, M. Szleper, P. Traczyk, P. Zalewski, K. Bunkowski, A. Byszuk, K. Doroba, A. Kalinowski, M. Konecki, J. Krolikowski, M. Misiura, M. Olszewski, A. Pyskir, M. Walczak, M. Araujo, P. Bargassa, D. Bastos, C. Beirão Da Cruz E Silva, A. Di Francesco, P. Faccioli, B. Galinhas, M. Gallinaro, J. Hollar, N. Leonardo, J. Seixas, G. Strong, O. Toldaiev, J. Varela, S. Afanasiev, P. Bunin, M. Gavrilenko, I. Golutvin, I. Gorbunov, A. Kamenev, V. Karjavine, A. Lanev, A. Malakhov, V. Matveev, P. Moisenz, V. Palichik, V. Perelygin, S. Shmatov, S. Shulha, N. Skatchkov, V. Smirnov, N. Voytishin, A. Zarubin, V. Golovtsov, Y. Ivanov, V. Kim, E. Kuznetsova, P. Levchenko, V. Murzin, V. Oreshkin, I. Smirnov, D. Sosnov, V. Sulimov, L. Uvarov, S. Vavilov, A. Vorobyev, Yu. Andreev, A. Dermenev, S. Gninenko, N. Golubev, A. Karneyeu, M. Kirsanov, N. Krasnikov, A. Pashenkov, A. Shabanov, D. Tlisov, A. Toropin, V. Epshteyn, V. Gavrilov, N. Lychkovskaya, V. Popov, I. Pozdnyakov, G. Safronov, A. Spiridonov, A. Stepennov, V. Stolin, M. Toms, E. Vlasov, A. Zhokin, T. Aushev, M. Chadeeva, D. Philippov, E. Popova, V. Rusinov, V. Andreev, M. Azarkin, I. Dremin, M. Kirakosyan, A. Terkulov, A. Belyaev, E. Boos, M. Dubinin, L. Dudko, A. Ershov, A. Gribushin, V. Klyukhin, O. Kodolova, I. Lokhtin, S. Obraztsov, S. Petrushanko, V. Savrin, A. Snigirev, A. Barnyakov, V. Blinov, T. Dimova, L. Kardapoltsev, Y. Skovpen, I. Azhgirey, I. Bayshev, S. Bitioukov, V. Kachanov, A. Kalinin, D. Konstantinov, P. Mandrik, V. Petrov, R. Ryutin, S. Slabospitskii, A. Sobol, S. Troshin, N. Tyurin, A. Uzunian, A. Volkov, A. Babaev, S. Baidali, A. Iuzhakov, V. Okhotnikov, P. Adzic, P. Cirkovic, D. Devetak, M. Dordevic, P. Milenovic, J. Milosevic, J. Alcaraz Maestre, A. lvarez Fernández, I. Bachiller, M. Barrio Luna, J. A. Brochero Cifuentes, M. Cerrada, N. Colino, B. De La Cruz, A. Delgado Peris, C. Fernandez Bedoya, J. P. Fernández Ramos, J. Flix, M. C. Fouz, O. Gonzalez Lopez, S. Goy Lopez, J. M. Hernandez, M. I. Josa, D. Moran, A. Pérez-Calero Yzquierdo, J. Puerta Pelayo, I. Redondo, L. Romero, S. Sánchez Navas, M. S. Soares, A. Triossi, C. Albajar, J. F. de Trocóniz, J. Cuevas, C. Erice, J. Fernandez Menendez, S. Folgueras, I. Gonzalez Caballero, J. R. González Fernández, E. Palencia Cortezon, V. Rodríguez Bouza, S. Sanchez Cruz, J. M. Vizan Garcia, I. J. Cabrillo, A. Calderon, B. Chazin Quero, J. Duarte Campderros, M. Fernandez, P. J. Fernández Manteca, A. García Alonso, G. Gomez, A. Lopez Virto, C. Martinez Rivero, P. Martinez Ruiz del Arbol, F. Matorras, J. Piedra Gomez, C. Prieels, T. Rodrigo, A. Ruiz-Jimeno, L. Scodellaro, N. Trevisani, I. Vila, N. Wickramage, D. Abbaneo, B. Akgun, E. Auffray, G. Auzinger, P. Baillon, A. H. Ball, D. Barney, J. Bendavid, M. Bianco, A. Bocci, C. Botta, E. Brondolin, T. Camporesi, M. Cepeda, G. Cerminara, E. Chapon, Y. Chen, G. Cucciati, D. d’Enterria, A. Dabrowski, N. Daci, V. Daponte, A. David, A. De Roeck, N. Deelen, M. Dobson, M. Dünser, N. Dupont, A. Elliott-Peisert, F. Fallavollita, D. Fasanella, G. Franzoni, J. Fulcher, W. Funk, D. Gigi, A. Gilbert, K. Gill, F. Glege, M. Gruchala, M. Guilbaud, D. Gulhan, J. Hegeman, C. Heidegger, Y. Iiyama, V. Innocente, G. M. Innocenti, A. Jafari, P. Janot, O. Karacheban, J. Kieseler, A. Kornmayer, M. Krammer, C. Lange, P. Lecoq, C. Lourenço, L. Malgeri, M. Mannelli, A. Massironi, F. Meijers, J. A. Merlin, S. Mersi, E. Meschi, F. Moortgat, M. Mulders, J. Ngadiuba, S. Nourbakhsh, S. Orfanelli, L. Orsini, F. Pantaleo, L. Pape, E. Perez, M. Peruzzi, A. Petrilli, G. Petrucciani, A. Pfeiffer, M. Pierini, F. M. Pitters, D. Rabady, A. Racz, M. Rovere, H. Sakulin, C. Schäfer, C. Schwick, M. Selvaggi, A. Sharma, P. Silva, P. Sphicas, A. Stakia, J. Steggemann, V. R. Tavolaro, D. Treille, A. Tsirou, A. Vartak, M. Verzetti, W. D. Zeuner, L. Caminada, K. Deiters, W. Erdmann, R. Horisberger, Q. Ingram, H. C. Kaestli, D. Kotlinski, U. Langenegger, T. Rohe, S. A. Wiederkehr, M. Backhaus, P. Berger, N. Chernyavskaya, G. Dissertori, M. Dittmar, M. Donegà, C. Dorfer, T. A. Gómez Espinosa, C. Grab, D. Hits, T. Klijnsma, W. Lustermann, R. A. Manzoni, M. Marionneau, M. T. Meinhard, F. Micheli, P. Musella, F. Nessi-Tedaldi, F. Pauss, G. Perrin, L. Perrozzi, S. Pigazzini, M. Reichmann, C. Reissel, T. Reitenspiess, D. Ruini, D. A. Sanz Becerra, M. Schönenberger, L. Shchutska, K. Theofilatos, M. L. Vesterbacka Olsson, R. Wallny, D. H. Zhu, T. K. Aarrestad, C. Amsler, D. Brzhechko, M. F. Canelli, A. De Cosa, R. Del Burgo, S. Donato, C. Galloni, T. Hreus, B. Kilminster, S. Leontsinis, V. M. Mikuni, I. Neutelings, G. Rauco, P. Robmann, D. Salerno, K. Schweiger, C. Seitz, Y. Takahashi, S. Wertz, A. Zucchetta, T. H. Doan, C. M. Kuo, W. Lin, S. S. Yu, P. Chang, Y. Chao, K. F. Chen, P. H. Chen, W.-S. Hou, Y. F. Liu, R.-S. Lu, E. Paganis, A. Psallidas, A. Steen, B. Asavapibhop, N. Srimanobhas, N. Suwonjandee, A. Bat, F. Boran, S. Cerci, S. Damarseckin, Z. S. Demiroglu, F. Dolek, C. Dozen, I. Dumanoglu, G. Gokbulut, EmineGurpinar Guler, Y. Guler, I. Hos, C. Isik, E. E. Kangal, O. Kara, A. Kayis Topaksu, U. Kiminsu, M. Oglakci, G. Onengut, K. Ozdemir, S. Ozturk, D. Sunar Cerci, B. Tali, U. G. Tok, S. Turkcapar, I. S. Zorbakir, C. Zorbilmez, B. Isildak, G. Karapinar, M. Yalvac, M. Zeyrek, I. O. Atakisi, E. Gülmez, M. Kaya, O. Kaya, B. Kaynak, Ö. Özçelik, S. Ozkorucuklu, S. Tekten, E. A. Yetkin, A. Cakir, K. Cankocak, Y. Komurcu, S. Sen, B. Grynyov, L. Levchuk, F. Ball, J. J. Brooke, D. Burns, E. Clement, D. Cussans, O. Davignon, H. Flacher, J. Goldstein, G. P. Heath, H. F. Heath, L. Kreczko, D. M. Newbold, S. Paramesvaran, B. Penning, T. Sakuma, D. Smith, V. J. Smith, J. Taylor, A. Titterton, K. W. Bell, A. Belyaev, C. Brew, R. M. Brown, D. Cieri, D. J. A. Cockerill, J. A. Coughlan, K. Harder, S. Harper, J. Linacre, K. Manolopoulos, E. Olaiya, D. Petyt, T. Reis, T. Schuh, C. H. Shepherd-Themistocleous, A. Thea, I. R. Tomalin, T. Williams, W. J. Womersley, R. Bainbridge, P. Bloch, J. Borg, S. Breeze, O. Buchmuller, A. Bundock, GurpreetSingh CHAHAL, D. Colling, P. Dauncey, G. Davies, M. Della Negra, R. Di Maria, P. Everaerts, G. Hall, G. Iles, T. James, M. Komm, C. Laner, L. Lyons, A.-M. Magnan, S. Malik, A. Martelli, V. Milosevic, J. Nash, A. Nikitenko, V. Palladino, M. Pesaresi, D. M. Raymond, A. Richards, A. Rose, E. Scott, C. Seez, A. Shtipliyski, M. Stoye, T. Strebler, S. Summers, A. Tapper, K. Uchida, T. Virdee, N. Wardle, D. Winterbottom, J. Wright, S. C. Zenz, J. E. Cole, P. R. Hobson, A. Khan, P. Kyberd, C. K. Mackay, A. Morton, I. D. Reid, L. Teodorescu, S. Zahid, K. Call, J. Dittmann, K. Hatakeyama, C. Madrid, B. McMaster, N. Pastika, C. Smith, R. Bartek, A. Dominguez, A. Buccilli, O. Charaf, S. I. Cooper, C. Henderson, P. Rumerio, C. West, D. Arcaro, T. Bose, Z. Demiragli, D. Gastler, S. Girgis, D. Pinna, C. Richardson, J. Rohlf, D. Sperka, I. Suarez, L. Sulak, D. Zou, G. Benelli, B. Burkle, X. Coubez, D. Cutts, M. Hadley, J. Hakala, U. Heintz, J. M. Hogan, K. H. M. Kwok, E. Laird, G. Landsberg, J. Lee, Z. Mao, M. Narain, S. Sagir, R. Syarif, E. Usai, D. Yu, R. Band, C. Brainerd, R. Breedon, D. Burns, M. Calderon De La Barca Sanchez, M. Chertok, J. Conway, R. Conway, P. T. Cox, R. Erbacher, C. Flores, G. Funk, W. Ko, O. Kukral, R. Lander, M. Mulhearn, D. Pellett, J. Pilot, M. Shi, D. Stolp, D. Taylor, K. Tos, M. Tripathi, Z. Wang, F. Zhang, M. Bachtis, C. Bravo, R. Cousins, A. Dasgupta, A. Florent, J. Hauser, M. Ignatenko, N. Mccoll, S. Regnard, D. Saltzberg, C. Schnaible, V. Valuev, K. Burt, R. Clare, J. W. Gary, S. M. A. Ghiasi Shirazi, G. Hanson, G. Karapostoli, E. Kennedy, O. R. Long, M. Olmedo Negrete, M. I. Paneva, W. Si, L. Wang, H. Wei, S. Wimpenny, B. R. Yates, J. G. Branson, P. Chang, S. Cittolin, M. Derdzinski, R. Gerosa, D. Gilbert, B. Hashemi, A. Holzner, D. Klein, G. Kole, V. Krutelyov, J. Letts, M. Masciovecchio, S. May, D. Olivito, S. Padhi, M. Pieri, V. Sharma, M. Tadel, J. Wood, F. Würthwein, A. Yagil, G. Zevi Della Porta, N. Amin, R. Bhandari, C. Campagnari, M. Citron, V. Dutta, M. Franco Sevilla, L. Gouskos, J. Incandela, B. Marsh, H. Mei, A. Ovcharova, H. Qu, J. Richman, U. Sarica, D. Stuart, S. Wang, J. Yoo, D. Anderson, A. Bornheim, J. M. Lawhorn, N. Lu, H. B. Newman, T. Q. Nguyen, J. Pata, M. Spiropulu, J. R. Vlimant, R. Wilkinson, S. Xie, Z. Zhang, R. Y. Zhu, M. B. Andrews, T. Ferguson, T. Mudholkar, M. Paulini, M. Sun, I. Vorobiev, M. Weinberg, J. P. Cumalat, W. T. Ford, F. Jensen, A. Johnson, E. MacDonald, T. Mulholland, R. Patel, A. Perloff, K. Stenson, K. A. Ulmer, S. R. Wagner, J. Alexander, J. Chaves, Y. Cheng, J. Chu, A. Datta, A. Frankenthal, K. Mcdermott, N. Mirman, J. Monroy, J. R. Patterson, D. Quach, A. Rinkevicius, A. Ryd, L. Skinnari, L. Soffi, S. M. Tan, Z. Tao, J. Thom, J. Tucker, P. Wittich, M. Zientek, S. Abdullin, M. Albrow, M. Alyari, G. Apollinari, A. Apresyan, A. Apyan, S. Banerjee, L. A. T. Bauerdick, A. Beretvas, J. Berryhill, P. C. Bhat, K. Burkett, J. N. Butler, A. Canepa, G. B. Cerati, H. W. K. Cheung, F. Chlebana, M. Cremonesi, J. Duarte, V. D. Elvira, J. Freeman, Z. Gecse, E. Gottschalk, L. Gray, D. Green, S. Grünendahl, O. Gutsche, J. Hanlon, R. M. Harris, S. Hasegawa, R. Heller, J. Hirschauer, Z. Hu, B. Jayatilaka, S. Jindariani, M. Johnson, U. Joshi, B. Klima, M. J. Kortelainen, B. Kreis, S. Lammel, D. Lincoln, R. Lipton, M. Liu, T. Liu, J. Lykken, K. Maeshima, J. M. Marraffino, D. Mason, P. McBride, P. Merkel, S. Mrenna, S. Nahn, V. O’Dell, K. Pedro, C. Pena, O. Prokofyev, G. Rakness, F. Ravera, A. Reinsvold, L. Ristori, B. Schneider, E. Sexton-Kennedy, N. Smith, A. Soha, W. J. Spalding, L. Spiegel, S. Stoynev, J. Strait, N. Strobbe, L. Taylor, S. Tkaczyk, N. V. Tran, L. Uplegger, E. W. Vaandering, C. Vernieri, M. Verzocchi, R. Vidal, M. Wang, H. A. Weber, D. Acosta, P. Avery, P. Bortignon, D. Bourilkov, A. Brinkerhoff, L. Cadamuro, A. Carnes, V. Cherepanov, D. Curry, R. D. Field, S. V. Gleyzer, B. M. Joshi, M. Kim, J. Konigsberg, A. Korytov, K. H. Lo, P. Ma, K. Matchev, N. Menendez, G. Mitselmakher, D. Rosenzweig, K. Shi, J. Wang, S. Wang, X. Zuo, Y. R. Joshi, S. Linn, T. Adams, A. Askew, S. Hagopian, V. Hagopian, K. F. Johnson, R. Khurana, T. Kolberg, G. Martinez, T. Perry, H. Prosper, A. Saha, C. Schiber, R. Yohay, M. M. Baarmand, V. Bhopatkar, S. Colafranceschi, M. Hohlmann, D. Noonan, M. Rahmani, T. Roy, M. Saunders, F. Yumiceva, M. R. Adams, L. Apanasevich, D. Berry, R. R. Betts, R. Cavanaugh, X. Chen, S. Dittmer, O. Evdokimov, C. E. Gerber, D. A. Hangal, D. J. Hofman, K. Jung, C. Mills, M. B. Tonjes, N. Varelas, H. Wang, X. Wang, Z. Wu, J. Zhang, M. Alhusseini, B. Bilki, W. Clarida, K. Dilsiz, S. Durgut, R. P. Gandrajula, M. Haytmyradov, V. Khristenko, O. K. Köseyan, J.-P. Merlo, A. Mestvirishvili, A. Moeller, J. Nachtman, H. Ogul, Y. Onel, F. Ozok, A. Penzo, C. Snyder, E. Tiras, J. Wetzel, B. Blumenfeld, A. Cocoros, N. Eminizer, D. Fehling, L. Feng, A. V. Gritsan, W. T. Hung, P. Maksimovic, J. Roskes, M. Swartz, M. Xiao, A. Al-bataineh, P. Baringer, A. Bean, S. Boren, J. Bowen, A. Bylinkin, J. Castle, S. Khalil, A. Kropivnitskaya, D. Majumder, W. Mcbrayer, M. Murray, C. Rogan, S. Sanders, E. Schmitz, J. D. Tapia Takaki, Q. Wang, S. Duric, A. Ivanov, K. Kaadze, D. Kim, Y. Maravin, D. R. Mendis, T. Mitchell, A. Modak, A. Mohammadi, F. Rebassoo, D. Wright, A. Baden, O. Baron, A. Belloni, S. C. Eno, Y. Feng, C. Ferraioli, N. J. Hadley, S. Jabeen, G. Y. Jeng, R. G. Kellogg, J. Kunkle, A. C. Mignerey, S. Nabili, F. Ricci-Tam, M. Seidel, Y. H. Shin, A. Skuja, S. C. Tonwar, K. Wong, D. Abercrombie, B. Allen, V. Azzolini, A. Baty, R. Bi, S. Brandt, W. Busza, I. A. Cali, M. D’Alfonso, G. Gomez Ceballos, M. Goncharov, P. Harris, D. Hsu, M. Hu, M. Klute, D. Kovalskyi, Y.-J. Lee, P. D. Luckey, B. Maier, A. C. Marini, C. Mcginn, C. Mironov, S. Narayanan, X. Niu, C. Paus, D. Rankin, C. Roland, G. Roland, Z. Shi, G. S. F. Stephans, K. Sumorok, K. Tatar, D. Velicanu, J. Wang, T. W. Wang, B. Wyslouch, A. C. Benvenuti, R. M. Chatterjee, A. Evans, P. Hansen, J. Hiltbrand, Sh. Jain, S. Kalafut, M. Krohn, Y. Kubota, Z. Lesko, J. Mans, R. Rusack, M. A. Wadud, J. G. Acosta, S. Oliveros, E. Avdeeva, K. Bloom, D. R. Claes, C. Fangmeier, L. Finco, F. Golf, R. Gonzalez Suarez, R. Kamalieddin, I. Kravchenko, J. E. Siado, G. R. Snow, B. Stieger, A. Godshalk, C. Harrington, I. Iashvili, A. Kharchilava, C. Mclean, D. Nguyen, A. Parker, S. Rappoccio, B. Roozbahani, G. Alverson, E. Barberis, C. Freer, Y. Haddad, A. Hortiangtham, G. Madigan, D. M. Morse, T. Orimoto, A. Tishelman-Charny, T. Wamorkar, B. Wang, A. Wisecarver, D. Wood, S. Bhattacharya, J. Bueghly, T. Gunter, K. A. Hahn, N. Odell, M. H. Schmitt, K. Sung, M. Trovato, M. Velasco, R. Bucci, N. Dev, R. Goldouzian, M. Hildreth, K. Hurtado Anampa, C. Jessop, D. J. Karmgard, K. Lannon, W. Li, N. Loukas, N. Marinelli, F. Meng, C. Mueller, Y. Musienko, M. Planer, R. Ruchti, P. Siddireddy, G. Smith, S. Taroni, M. Wayne, A. Wightman, M. Wolf, A. Woodard, J. Alimena, L. Antonelli, B. Bylsma, L. S. Durkin, S. Flowers, B. Francis, C. Hill, W. Ji, A. Lefeld, T. Y. Ling, W. Luo, B. L. Winer, S. Cooperstein, G. Dezoort, P. Elmer, J. Hardenbrook, N. Haubrich, S. Higginbotham, A. Kalogeropoulos, S. Kwan, D. Lange, M. T. Lucchini, J. Luo, D. Marlow, K. Mei, I. Ojalvo, J. Olsen, C. Palmer, P. Piroué, J. Salfeld-Nebgen, D. Stickland, C. Tully, Z. Wang, S. Malik, S. Norberg, A. Barker, V. E. Barnes, S. Das, L. Gutay, M. Jones, A. W. Jung, A. Khatiwada, B. Mahakud, D. H. Miller, G. Negro, N. Neumeister, C. C. Peng, S. Piperov, H. Qiu, J. F. Schulte, J. Sun, F. Wang, R. Xiao, W. Xie, T. Cheng, J. Dolen, N. Parashar, Z. Chen, K. M. Ecklund, S. Freed, F. J. M. Geurts, M. Kilpatrick, Arun Kumar, W. Li, B. P. Padley, J. Roberts, J. Rorie, W. Shi, A. G. Stahl Leiton, Z. Tu, A. Zhang, A. Bodek, P. de Barbaro, R. Demina, Y. t. Duh, J. L. Dulemba, C. Fallon, T. Ferbel, M. Galanti, A. Garcia-Bellido, J. Han, O. Hindrichs, A. Khukhunaishvili, E. Ranken, P. Tan, R. Taus, B. Chiarito, J. P. Chou, Y. Gershtein, E. Halkiadakis, A. Hart, M. Heindl, E. Hughes, S. Kaplan, S. Kyriacou, I. Laflotte, A. Lath, R. Montalvo, K. Nash, M. Osherson, H. Saka, S. Salur, S. Schnetzer, D. Sheffield, S. Somalwar, R. Stone, S. Thomas, P. Thomassen, H. Acharya, A. G. Delannoy, J. Heideman, G. Riley, S. Spanier, O. Bouhali, A. Celik, M. Dalchenko, M. De Mattia, A. Delgado, S. Dildick, R. Eusebi, J. Gilmore, T. Huang, T. Kamon, S. Luo, D. Marley, R. Mueller, D. Overton, L. Perniè, D. Rathjens, A. Safonov, N. Akchurin, J. Damgov, F. De Guio, P. R. Dudero, S. Kunori, K. Lamichhane, S. W. Lee, T. Mengke, S. Muthumuni, T. Peltola, S. Undleeb, I. Volobouev, Z. Wang, A. Whitbeck, S. Greene, A. Gurrola, R. Janjam, W. Johns, C. Maguire, A. Melo, H. Ni, K. Padeken, F. Romeo, P. Sheldon, S. Tuo, J. Velkovska, M. Verweij, Q. Xu, M. W. Arenton, P. Barria, B. Cox, R. Hirosky, M. Joyce, A. Ledovskoy, H. Li, C. Neu, Y. Wang, E. Wolfe, F. Xia, R. Harr, P. E. Karchin, N. Poudyal, J. Sturdy, P. Thapa, S. Zaleski, J. Buchanan, C. Caillol, D. Carlsmith, S. Dasu, I. De Bruyn, L. Dodd, B. Gomber, M. Grothe, M. Herndon, A. Hervé, U. Hussain, P. Klabbers, A. Lanaro, K. Long, R. Loveless, T. Ruggles, A. Savin, V. Sharma, W. H. Smith, N. Woods, CMS Collaboration

**Affiliations:** 1 Yerevan Physics Institute, Yerevan, Armenia; 2 Institut für Hochenergiephysik, Wien, Austria; 3 Institute for Nuclear Problems, Minsk, Belarus; 4 Universiteit Antwerpen, Antwerp, Belgium; 5 Vrije Universiteit Brussel, Brussels, Belgium; 6 Université Libre de Bruxelles, Bruxelles, Belgium; 7 Ghent University, Ghent, Belgium; 8 Université Catholique de Louvain, Louvain-la-Neuve, Belgium; 9 Centro Brasileiro de Pesquisas Fisicas, Rio de Janeiro, Brazil; 10 Universidade do Estado do Rio de Janeiro, Rio de Janeiro, Brazil; 11 Universidade Estadual Paulista, Universidade Federal do ABC, São Paulo, Brazil; 12 Institute for Nuclear Research and Nuclear Energy, Bulgarian Academy of Sciences, Sofia, Bulgaria; 13 University of Sofia, Sofia, Bulgaria; 14 Beihang University, Beijing, China; 15 Institute of High Energy Physics, Beijing, China; 16 State Key Laboratory of Nuclear Physics and Technology, Peking University, Beijing, China; 17 Tsinghua University, Beijing, China; 18 Universidad de Los Andes, Bogota, Colombia; 19 Universidad de Antioquia, Medellin, Colombia; 20 University of Split, Faculty of Electrical Engineering, Mechanical Engineering and Naval Architecture, Split, Croatia; 21 University of Split, Faculty of Science, Split, Croatia; 22 Institute Rudjer Boskovic, Zagreb, Croatia; 23 University of Cyprus, Nicosia, Cyprus; 24 Charles University, Prague, Czech Republic; 25 Escuela Politecnica Nacional, Quito, Ecuador; 26 Universidad San Francisco de Quito, Quito, Ecuador; 27 Academy of Scientific Research and Technology of the Arab Republic of Egypt, Egyptian Network of High Energy Physics, Cairo, Egypt; 28 National Institute of Chemical Physics and Biophysics, Tallinn, Estonia; 29 Department of Physics, University of Helsinki, Helsinki, Finland; 30 Helsinki Institute of Physics, Helsinki, Finland; 31 Lappeenranta University of Technology, Lappeenranta, Finland; 32 IRFU, CEA, Université Paris-Saclay, Gif-sur-Yvette, France; 33 Laboratoire Leprince-Ringuet, CNRS/IN2P3, Ecole Polytechnique, Institut Polytechnique de Paris, Palaiseau, France; 34 Université de Strasbourg, CNRS, IPHC UMR 7178, Strasbourg, France; 35 Centre de Calcul de l’Institut National de Physique Nucleaire et de Physique des Particules, CNRS/IN2P3, Villeurbanne, France; 36 Université de Lyon, Université Claude Bernard Lyon 1, CNRS-IN2P3, Institut de Physique Nucléaire de Lyon, Villeurbanne, France; 37 Georgian Technical University, Tbilisi, Georgia; 38 Tbilisi State University, Tbilisi, Georgia; 39 RWTH Aachen University, I. Physikalisches Institut, Aachen, Germany; 40 RWTH Aachen University, III. Physikalisches Institut A, Aachen, Germany; 41 RWTH Aachen University, III. Physikalisches Institut B, Aachen, Germany; 42 Deutsches Elektronen-Synchrotron, Hamburg, Germany; 43 University of Hamburg, Hamburg, Germany; 44 Karlsruher Institut fuer Technologie, Karlsruhe, Germany; 45 Institute of Nuclear and Particle Physics (INPP), NCSR Demokritos, Aghia Paraskevi, Greece; 46 National and Kapodistrian University of Athens, Athens, Greece; 47 National Technical University of Athens, Athens, Greece; 48 University of Ioánnina, Ioánnina, Greece; 49 MTA-ELTE Lendület CMS Particle and Nuclear Physics Group, Eötvös Loránd University, Budapest, Hungary; 50 Wigner Research Centre for Physics, Budapest, Hungary; 51 Institute of Nuclear Research ATOMKI, Debrecen, Hungary; 52 Institute of Physics, University of Debrecen, Debrecen, Hungary; 53 Indian Institute of Science (IISc), Bangalore, India; 54 National Institute of Science Education and Research, HBNI, Bhubaneswar, India; 55 Panjab University, Chandigarh, India; 56 University of Delhi, Delhi, India; 57 Saha Institute of Nuclear Physics, HBNI, Kolkata, India; 58 Indian Institute of Technology Madras, Madras, India; 59 Bhabha Atomic Research Centre, Mumbai, India; 60 Tata Institute of Fundamental Research-A, Mumbai, India; 61 Tata Institute of Fundamental Research-B, Mumbai, India; 62 Indian Institute of Science Education and Research (IISER), Pune, India; 63 Institute for Research in Fundamental Sciences (IPM), Tehran, Iran; 64 University College Dublin, Dublin, Ireland; 65 INFN Sezione di Bari, Università di Bari, Politecnico di Bari, Bari, Italy; 66 INFN Sezione di Bologna, Università di Bologna, Bologna, Italy; 67 INFN Sezione di Catania, Università di Catania, Catania, Italy; 68 INFN Sezione di Firenze, Università di Firenze, Firenze, Italy; 69 INFN Laboratori Nazionali di Frascati, Frascati, Italy; 70 INFN Sezione di Genova, Università di Genova, Genova, Italy; 71 INFN Sezione di Milano-Bicocca, Università di Milano-Bicocca, Milan, Italy; 72 INFN Sezione di Napoli, Università di Napoli ’Federico II’ , Naples, Italy, Università della Basilicata, Potenza, Italy, Università G. Marconi, Rome, Italy; 73 INFN Sezione di Padova, Università di Padova, Padova, Italy, Università di Trento, Trento, Italy; 74 INFN Sezione di Pavia, Università di Pavia, Pavia, Italy; 75 INFN Sezione di Perugia, Università di Perugia, Perugia, Italy; 76 INFN Sezione di Pisa, Università di Pisa, Scuola Normale Superiore di Pisa, Pisa, Italy; 77 INFN Sezione di Roma, Sapienza Università di Roma, Rome, Italy; 78 INFN Sezione di Torino, Università di Torino, Torino, Italy, Università del Piemonte Orientale, Novara, Italy; 79 INFN Sezione di Trieste, Università di Trieste, Trieste, Italy; 80 Kyungpook National University, Taegu, Korea; 81 Chonnam National University, Institute for Universe and Elementary Particles, Kwangju, Korea; 82 Hanyang University, Seoul, Korea; 83 Korea University, Seoul, Korea; 84 Sejong University, Seoul, Korea; 85 Seoul National University, Seoul, Korea; 86 University of Seoul, Seoul, Korea; 87 Sungkyunkwan University, Suwon, Korea; 88 Riga Technical University, Riga, Latvia; 89 Vilnius University, Vilnius, Lithuania; 90 National Centre for Particle Physics, Universiti Malaya, Kuala Lumpur, Malaysia; 91 Universidad de Sonora (UNISON), Hermosillo, Mexico; 92 Centro de Investigacion y de Estudios Avanzados del IPN, Mexico, Mexico; 93 Universidad Iberoamericana, Mexico, Mexico; 94 Benemerita Universidad Autonoma de Puebla, Puebla, Mexico; 95 Universidad Autónoma de San Luis Potosí, San Luis Potosí, Mexico; 96 University of Montenegro, Podgorica, Montenegro; 97 University of Auckland, Auckland, New Zealand; 98 University of Canterbury, Christchurch, New Zealand; 99 National Centre for Physics, Quaid-I-Azam University, Islamabad, Pakistan; 100 National Centre for Nuclear Research, Swierk, Poland; 101 Institute of Experimental Physics, Faculty of Physics, University of Warsaw, Warsaw, Poland; 102 Laboratório de Instrumentação e Física Experimental de Partículas, Lisbon, Portugal; 103 Joint Institute for Nuclear Research, Dubna, Russia; 104 Petersburg Nuclear Physics Institute, Gatchina (St. Petersburg), Russia; 105 Institute for Nuclear Research, Moscow, Russia; 106 Institute for Theoretical and Experimental Physics named by A.I. Alikhanov of NRC ‘Kurchatov Institute’, Moscow, Russia; 107 Moscow Institute of Physics and Technology, Moscow, Russia; 108 National Research Nuclear University ’Moscow Engineering Physics Institute’ (MEPhI), Moscow, Russia; 109 P.N. Lebedev Physical Institute, Moscow, Russia; 110 Skobeltsyn Institute of Nuclear Physics, Lomonosov Moscow State University, Moscow, Russia; 111 Novosibirsk State University (NSU), Novosibirsk, Russia; 112 Institute for High Energy Physics of National Research Centre ‘Kurchatov Institute’, Protvino, Russia; 113 National Research Tomsk Polytechnic University, Tomsk, Russia; 114 University of Belgrade: Faculty of Physics and VINCA Institute of Nuclear Sciences, Beograde, Serbia; 115 Centro de Investigaciones Energéticas Medioambientales y Tecnológicas (CIEMAT), Madrid, Spain; 116 Universidad Autónoma de Madrid, Madrid, Spain; 117 Universidad de Oviedo, Instituto Universitario de Ciencias y Tecnologías Espaciales de Asturias (ICTEA), Oviedo, Spain; 118 Instituto de Física de Cantabria (IFCA), CSIC-Universidad de Cantabria, Santander, Spain; 119 Department of Physics, University of Ruhuna, Matara, Sri Lanka; 120 CERN, European Organization for Nuclear Research, Geneva, Switzerland; 121 Paul Scherrer Institut, Villigen, Switzerland; 122 ETH Zurich-Institute for Particle Physics and Astrophysics (IPA), Zurich, Switzerland; 123 Universität Zürich, Zurich, Switzerland; 124 National Central University, Chung-Li, Taiwan; 125 National Taiwan University (NTU), Taipei, Taiwan; 126 Department of Physics, Faculty of Science, Chulalongkorn University, Bangkok, Thailand; 127 Physics Department, Science and Art Faculty, Ukurova University, Adana, Turkey; 128 Middle East Technical University, Physics Department, Ankara, Turkey; 129 Bogazici University, Istanbul, Turkey; 130 Istanbul Technical University, Istanbul, Turkey; 131 Institute for Scintillation Materials of National Academy of Science of Ukraine, Kharkov, Ukraine; 132 National Scientific Center, Kharkov Institute of Physics and Technology, Kharkov, Ukraine; 133 University of Bristol, Bristol, UK; 134 Rutherford Appleton Laboratory, Didcot, UK; 135 Imperial College, London, UK; 136 Brunel University, Uxbridge, UK; 137 Baylor University, Waco, USA; 138 Catholic University of America, Washington, DC, USA; 139 The University of Alabama, Tuscaloosa, USA; 140 Boston University, Boston, USA; 141 Brown University, Providence, USA; 142 University of California, Davis, Davis, USA; 143 University of California, Los Angeles, USA; 144 University of California, Riverside, Riverside, USA; 145 University of California, San Diego, La Jolla, USA; 146 University of California, Santa Barbara-Department of Physics, Santa Barbara, USA; 147 California Institute of Technology, Pasadena, USA; 148 Carnegie Mellon University, Pittsburgh, USA; 149 University of Colorado Boulder, Boulder, USA; 150 Cornell University, Ithaca, USA; 151 Fermi National Accelerator Laboratory, Batavia, USA; 152 University of Florida, Gainesville, USA; 153 Florida International University, Miami, USA; 154 Florida State University, Tallahassee, USA; 155 Florida Institute of Technology, Melbourne, USA; 156 University of Illinois at Chicago (UIC), Chicago, USA; 157 The University of Iowa, Iowa City, USA; 158 Johns Hopkins University, Baltimore, USA; 159 The University of Kansas, Lawrence, USA; 160 Kansas State University, Manhattan, USA; 161 Lawrence Livermore National Laboratory, Livermore, USA; 162 University of Maryland, College Park, USA; 163 Massachusetts Institute of Technology, Cambridge, USA; 164 University of Minnesota, Minneapolis, USA; 165 University of Mississippi, Oxford, USA; 166 University of Nebraska-Lincoln, Lincoln, USA; 167 State University of New York at Buffalo, Buffalo, USA; 168 Northeastern University, Boston, USA; 169 Northwestern University, Evanston, USA; 170 University of Notre Dame, Notre Dame, USA; 171 The Ohio State University, Columbus, USA; 172 Princeton University, Princeton, USA; 173 University of Puerto Rico, Mayaguez, USA; 174 Purdue University, West Lafayette, USA; 175 Purdue University Northwest, Hammond, USA; 176 Rice University, Houston, USA; 177 University of Rochester, Rochester, USA; 178 Rutgers, The State University of New Jersey, Piscataway, USA; 179 University of Tennessee, Knoxville, USA; 180 Texas A&M University, College Station, USA; 181 Texas Tech University, Lubbock, USA; 182 Vanderbilt University, Nashville, USA; 183 University of Virginia, Charlottesville, USA; 184 Wayne State University, Detroit, USA; 185 University of Wisconsin-Madison, Madison, WI USA; 186 CERN, 1211 Geneva 23, Switzerland

## Abstract

A measurement is presented of electroweak (EW) production of a $\mathrm{W}$ boson in association with two jets in proton–proton collisions at $\sqrt{s}=13\,\text {Te}\text {V}$. The data sample was recorded by the CMS Collaboration at the LHC and corresponds to an integrated luminosity of 35.9$\,\text {fb}^{-1}$. The measurement is performed for the $\ell \nu$jj final state (with $\ell \nu$ indicating a lepton–neutrino pair, and j representing the quarks produced in the hard interaction) in a kinematic region defined by invariant mass $m_\mathrm {jj} >120\,\text {Ge}\text {V}$ and transverse momenta $p_\mathrm {T j} > 25\,\text {Ge}\text {V}$. The cross section of the process is measured in the electron and muon channels yielding $\sigma _\mathrm {EW}(\mathrm{W} \mathrm {jj})= 6.23 \pm 0.12 \,\text {(stat)} \pm 0.61 \,\text {(syst)} \,\text {pb}$ per channel, in agreement with leading-order standard model predictions. The additional hadronic activity of events in a signal-enriched region is studied, and the measurements are compared with predictions. The final state is also used to perform a search for anomalous trilinear gauge couplings. Limits on anomalous trilinear gauge couplings associated with dimension-six operators are given in the framework of an effective field theory. The corresponding 95% confidence level intervals are $-2.3< c_{{\mathrm{W} \mathrm{W} \mathrm{W}}}/\varLambda ^2 < 2.5\,\text {Te}\text {V} ^{-2}$, $-8.8< c_{\mathrm{W}}/\varLambda ^2 < 16\,\text {Te}\text {V} ^{-2}$, and $-45< c_{\mathrm{B}}/\varLambda ^2 < 46\,\text {Te}\text {V} ^{-2}$. These results are combined with the CMS EW $\mathrm{Zjj}$ analysis, yielding the constraint on the $c_{{\mathrm{W} \mathrm{W} \mathrm{W}}}$ coupling: $-1.8< c_{{\mathrm{W} \mathrm{W} \mathrm{W}}}/\varLambda ^2 < 2.0\,\text {Te}\text {V} ^{-2}$.

## Introduction

In proton–proton (pp) collisions at the CERN LHC, the pure electroweak (EW) production of a lepton–neutrino pair ($\ell \nu$) in association with two jets ($\mathrm {jj}$) includes production via vector boson fusion (VBF). This process has a distinctive signature of two jets with large energy and separation in pseudorapidity ($\eta$), produced in association with a lepton–neutrino pair. This EW process is referred to as EW $\mathrm{W} \mathrm {jj}$, and the two jets produced through the fragmentation of the outgoing quarks are referred to as “tagging jets”.

**Fig. 1:**
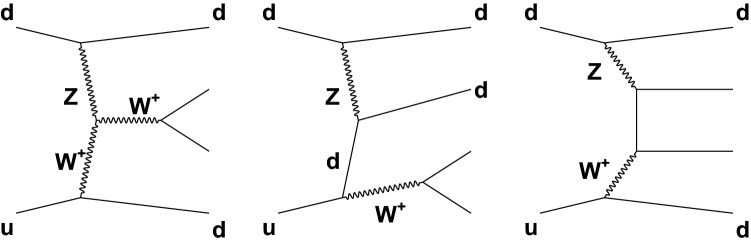
Representative Feynman diagrams for lepton–neutrino production in association with two jets from purely electroweak amplitudes: vector boson fusion (left), bremsstrahlung-like (center), and multiperipheral (right) production

Figure 1 shows representative Feynman diagrams for the EW $\mathrm{W} \mathrm {jj}$ signal processes, namely VBF (Fig. 1, left), bremsstrahlung-like (Fig. 1, center), and multiperipheral (Fig. 1, right) production. Gauge cancellations lead to a large negative interference between the VBF diagram and the other two diagrams, with the larger interference coming from bremsstrahlung-like production. Interference with multiperipheral production is limited to cases where the lepton–neutrino pair mass is close to the $\mathrm{W}$ boson mass.

**Fig. 2:**
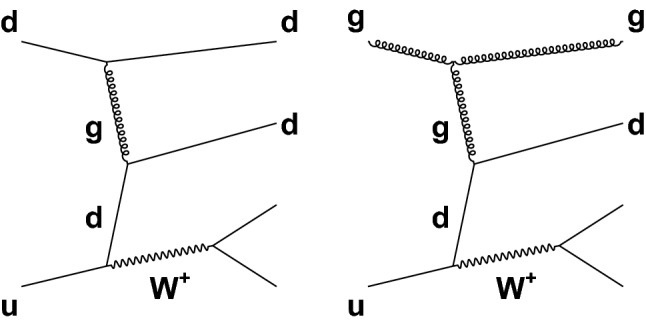
Representative diagrams for $\mathrm{W}$ boson production in association with two jets ($\mathrm {DY}\,\mathrm{W} \mathrm {jj}$) that constitute the main background for the measurement

In addition to the purely EW signal diagrams described above, there are other, not purely EW processes, that lead to the same $\ell \nu \mathrm {jj}$ final states and can interfere with the signal diagrams in Fig. 1. This interference effect between the signal production and the main Drell–Yan (DY) background processes ($\mathrm {DY}\,\mathrm{W} \mathrm {jj}$) is small compared to the interference effects among the EW production amplitudes, but needs to be included when measuring the signal contribution. Figure 2 (left) shows one example of $\mathrm{W}$ boson production in association with two jets that has the same initial and final states as those in Fig. 1. A process that does not interfere with the EW signal is shown in Fig. 2 (right).

The study of EW $\mathrm{W} \mathrm {jj}$ processes is part of a more general investigation of standard model (SM) VBF and scattering processes that includes the measurements of EW $\mathrm{Zjj}$ processes, Higgs boson production [1–3], and searches for physics beyond the SM [4]. The properties of EW $\mathrm{W} \mathrm {jj}$ events that are isolated from the backgrounds can be compared with SM predictions. Probing the additional hadronic activity in selected events can shed light on the modeling of the additional parton radiation [5, 6], which is important for signal selection and the vetoing of background events.

Higher-dimensional operators outside the SM can generate anomalous trilinear gauge couplings (ATGCs) [7, 8], so the measurement of the coupling strengths provides an indirect search for beyond-the-SM physics at mass scales not directly accessible at the LHC.

At the LHC, the EW $\mathrm{W} \mathrm {jj}$ process was first measured by the CMS Collaboration using pp collisions at $\sqrt{s}=8\,\text {Te}\text {V}$ [9] and then by the ATLAS Collaboration at both $\sqrt{s}=8\,\text {Te}\text {V}$ and $\sqrt{s}=7\,\text {Te}\text {V}$ [10]. The closely related EW $\mathrm{Zjj}$ process was first measured during Run 1 by the CMS Collaboration using pp collisions at $\sqrt{s}=7\,\text {Te}\text {V}$ [11], and then at $\sqrt{s}=8\,\text {Te}\text {V}$ by both the CMS [12] and ATLAS [13] Collaborations. The EW $\mathrm{Zjj}$ measurements using data samples of pp collisions at $\sqrt{s}=13\,\text {Te}\text {V}$ have been performed by ATLAS [14] and by CMS [15]. Considering leptonic final states in the same kinematic region the EW $\mathrm{W} \mathrm {jj}$ cross section is about a factor 10 larger than the EW $\mathrm{Zjj}$ cross section. All results so far agree with the expectations of the SM within a precision of 10–20%.

This paper presents measurements of the EW $\mathrm{W} \mathrm {jj}$ process with the CMS detector using pp collisions collected at $\sqrt{s}=$13$\,\text {Te}\text {V}$ during 2016, corresponding to an integrated luminosity of 35.9$\,\text {fb}^{-1}$. A multivariate analysis (BDT), based on the methods developed for the EW Zjj measurement [11, 12], is used to separate signal events from the large $\mathrm{W}$+jets background. The analysis of the 13$\,\text {Te}\text {V}$ data offers the opportunity to measure the cross section at a higher energy than previously done and to reduce the uncertainties obtained with previous measurements, given both the larger integrated luminosity and the larger predicted total cross section.

This paper is organized as follows: Sect. 2 describes the experimental apparatus and Sect. 3 the event simulations. Event selection procedures are described in Sect. 4, together with the selection efficiencies and background estimations using control regions (CRs). Section 5 describes an estimation of the multijet background from quantum chromodynamics (QCD), based on CRs in data. Section 6 discusses a correction applied to the simulation as a function of the invariant mass $m_\mathrm {jj}$. Section 7 presents distributions of the main discriminating variables in data. Section 8 details the strategy adopted to extract the signal from the data, and the corresponding systematic uncertainties are summarized in Sect. 9. The cross section and anomalous coupling results are presented in Sects. 10 and 11, respectively. Section 12 presents a study of the additional hadronic activity in an EW $\mathrm{W} \mathrm {jj}$ enriched region. Finally, a brief summary of the results is given in Sect. 13.

## The CMS detector and physics objects

The central feature of the CMS apparatus is a superconducting solenoid of 6$\,\text {m}$ internal diameter, providing a magnetic field of 3.8$\,\text {T}$. Within the solenoid volume are a silicon pixel and strip tracker, a lead tungstate crystal electromagnetic calorimeter (ECAL), and a brass and scintillator hadron calorimeter, each composed of a barrel and two endcap sections. Forward calorimeters extend the $\eta$ coverage provided by the barrel and endcap detectors to $|\eta |$ = 5.2. Muons are measured in gas-ionization detectors embedded in the steel flux-return yoke outside the solenoid.

The tracker measures charged particles within the range $|\eta | < 2.5$. It consists of 1440 pixel and 15,148 strip detector modules. For nonisolated particles with transverse momenta $1< p_{\mathrm {T}} < 10\,\text {Ge}\text {V}$ and $|\eta | < 1.4$, the track resolutions are typically 1.5% in $p_{\mathrm {T}}$ and 25–90 (45–150) $\upmu$m in the transverse (longitudinal) impact parameter [16].

The energy of electrons is measured after combining the information from the ECAL and the tracker, whereas their direction is measured by the tracker. The momentum resolution for electrons with $p_{\mathrm {T}} \approx$ 45$\,\text {Ge}\text {V}$ from ${\mathrm{Z}} \rightarrow \mathrm{e} \mathrm{e}$ decays ranges from 1.7 to 4.5%. It is generally better in the barrel region than in the endcaps, and also depends on the bremsstrahlung energy emitted by the electron as it traverses the material in front of the ECAL [17].

Muons are measured in the range $|\eta | < 2.4$, with detection planes made using three technologies: drift tubes, cathode strip chambers, and resistive-plate chambers. Matching muons to tracks measured in the silicon tracker results in a relative transverse momentum resolution for muons with $20<p_{\mathrm {T}} < 100\,\text {Ge}\text {V}$ of 1.3–2.0% in the barrel and better than 6% in the endcaps. The $p_{\mathrm {T}}$ resolution in the barrel is better than 10% for muons with $p_{\mathrm {T}}$ up to 1$\,\text {Te}\text {V}$ [18].

Events of interest are selected using a two-tiered trigger system [19]. The first level (L1), composed of custom hardware processors, uses information from the calorimeters and muon detectors to select events at a rate of around 100$\,\text {kHz}$ within a time interval of less than 4 $\upmu$s. The second level, known as the high-level trigger (HLT), consists of a farm of processors running a version of the full-event reconstruction software optimized for fast processing, and reduces the event rate to around 1$\,\text {kHz}$ before data storage.

A more detailed description of the CMS detector, together with a definition of the coordinate system used and the relevant kinematic variables, can be found in Ref. [20].

## Simulation of signal and background events

Signal events are simulated at leading order (LO) using the MadGraph 5_amc@nlo (v2.3.3) Monte Carlo (MC) generator [21], interfaced with pythia (v8.212) [22] for parton showering (PS) and hadronization. The NNPDF30 [23] parton distribution functions (PDFs) are used to generate the events. The underlying event is modeled using the CUETP8M1 tune [24]. The simulation does not include extra partons at matrix element (ME) level. The signal is defined in the kinematic region with parton transverse momentum $p_\mathrm {T j} > 25\,\text {Ge}\text {V}$, and diparton invariant mass $m_\mathrm {jj} >120\,\text {Ge}\text {V}$. The simulated cross section for the $\ell \nu$jj final state (with $\ell$ = e, $\mu$ or $\tau$), applying the above requirements, is $\sigma _\mathrm {LO}(\mathrm {EW}~\ell \nu \mathrm {jj})= 6.81 ^{+0.03}_{-0.06} \,\text {(scale)}\pm 0.26\,\text {(PDFs)}\,\text {pb}$, where the first uncertainty is obtained by changing simultaneously the factorization ($\mu _\mathrm {F}$) and renormalization ($\mu _{\mathrm {R}}$) scales by factors of 2 and 1/2, and the second one reflects the uncertainties in the NNPDF30 PDFs. The LO signal cross section and relevant kinematic distributions estimated with MadGraph 5_amc@nlo are in agreement within 2–5% with the next-to-leading-order (NLO) predictions of the vbfnlo generator (v2.6.3) [25–27], which include QCD NLO corrections to the LO ME-level diagrams evaluated with MadGraph 5_amc@nlo. For additional comparisons, signal events produced with MadGraph 5_amc@nlo are also processed with the herwig++ (v2.7.1) [28] PS, using the EE5C [29] tune.

An additional signal sample that includes NLO QCD corrections but does not include the s-channel contributions to the final state has been generated with powheg (v2.0) [30–32], based on the vbfnlo ME calculations [33, 34]. In the powheg sample the $m_\mathrm {jj} >120\,\text {Ge}\text {V}$ condition is applied on the two $p_{\mathrm {T}}$-leading parton-level jets, after clustering the ME final state partons with the $k_{\mathrm {T}}$-algorithm [35–37], with a distance parameter $D=0.8$, as done in Ref. [33]. The powheg sample has also been processed alternatively with pythia and herwig++ parton showering (PS) and hadronization programs, as done for the MadGraph 5_amc@nlo samples. In the following, results obtained with the powheg signal samples are given as a cross check of the main results obtained with the MadGraph 5_amc@nlo signal samples.

Events coming from processes including ATGCs are generated with the same settings as the SM sample, but include additional information for reweighting in the three-dimensional effective field theory (EFT) parameter space, which is described in more detail in Sect. 11. The ‘EWdim6NLO’ model [8, 21] is used for the generation of anomalous couplings.

Background $\mathrm{W}$ boson events are also simulated with MadGraph 5_amc@nlo using (1) an NLO ME calculation with up to three final-state partons generated from QCD interactions, and (2) an LO ME calculation with up to four partons from QCD interactions. The ME-PS matching is performed following the FxFx prescription [38] for the NLO case, and the MLM prescription [39, 40] for the LO case. The NLO background simulation is used to extract the final results, while the independent LO samples are used to perform the multivariate discriminant training. The inclusive $\mathrm{W}$ boson production is normalized to $\sigma _\text {th}(\mathrm{W})=61.5 \,\text {nb}$, as computed at next-to-next-to-leading order (NNLO) with fewz (v3.1) [41].

The evaluation of the interference between $\mathrm {EW}\,\mathrm{W} \mathrm {jj}$ and $\mathrm {DY}\,\mathrm{W} \mathrm {jj}$ processes relies on the predictions obtained with MadGraph 5_amc@nlo. A dedicated sample of events arising from the interference terms is generated directly by selecting the contributions of order $\alpha _\mathrm {s}\alpha _\mathrm {EW}^3$, and passed through the full detector simulation to estimate the expected interference contribution.

Other backgrounds are expected from events with one electron or muon and missing transverse momentum together with jets in the final state. Events from top quark pair production are generated with powheg (v2.0) [30–32], and normalized to the inclusive cross section calculated at NNLO, including next-to-next-to-leading logarithmic corrections, of 832$\,\text {pb}$ [42, 43]. Single top quark processes are modeled at NLO with powheg [30–32, 44] and normalized to cross sections of $71.7\pm 2.0\,\text {pb}$, $217\pm 3\,\text {pb}$, and $10.32\pm 0.20\,\text {pb}$, respectively, for the tW (powheg v1) [45], *t*-, and *s*-channel production [42, 46]. The diboson (VV) production processes ($\mathrm{W} \mathrm{W}$, $\mathrm{W} \mathrm{Z}$, and $\mathrm{Z} \mathrm{Z}$) are generated with pythia and normalized to NNLO cross section computations obtained with mcfm (v8.0) [47].

The contribution from QCD multijet processes is derived via an extrapolation from a QCD data CR with the lepton relative isolation selection inverted. All background simulations make use of the pythia PS model with the CUETP8M1 tune.

A detector simulation based on Geant4 (v9.4p03) [48, 49] is applied to all the generated signal and background samples. The presence of multiple pp interactions is incorporated by simulating additional interactions (pileup), both in-time and out-of-time with respect to the hard interaction, with a multiplicity that matches the distribution observed in data. The average pileup is measured to be about 23 additional interactions per bunch crossing.

## Reconstruction and selection of events

Events containing exactly one isolated, high-$p_{\mathrm {T}}$ lepton and at least two high-$p_{\mathrm {T}}$ jets are selected. Isolated single-lepton triggers are used to acquire the data, where the lepton is required to have $p_{\mathrm {T}} >27\,\text {Ge}\text {V}$ for the electron trigger and $p_{\mathrm {T}} >24\,\text {Ge}\text {V}$ for the muon trigger.

The offline analysis uses candidates reconstructed by the particle-flow (PF) algorithm [50]. In the PF event reconstruction, all stable particles in the event — i.e., electrons, muons, photons, charged and neutral hadrons — are reconstructed as PF candidates using information from all subdetectors to obtain an optimal determination of their direction, energy, and type. The PF candidates are used to reconstruct the jets and the missing transverse momentum.

The reconstructed primary vertex (PV) with the largest value of summed physics-object $p_{\mathrm {T}} ^2$ is the primary $\mathrm{p} \mathrm{p}$ interaction vertex. The physics objects are the objects returned by a jet finding algorithm [51, 52] applied to all charged particle tracks associated with the vertex, along with the corresponding associated missing transverse momentum. Charged tracks identified as hadrons from pileup vertices are omitted in the subsequent PF event reconstruction [50].

Offline electrons are reconstructed from clusters of energy deposits in the ECAL that match tracks extrapolated from the silicon tracker [17]. Offline muons are reconstructed by fitting trajectories based on hits in the silicon tracker and in the muon system [53]. Reconstructed electron or muon candidates are required to have $p_{\mathrm {T}} >20\,\text {Ge}\text {V}$. Electron candidates are required to be reconstructed within $|\eta |\le 2.4$, excluding the barrel-to-endcap transitional region $1.444< |\eta | < 1.566$ of the ECAL [20]. Muon candidates are required to be reconstructed in the fiducial region $|\eta |\le 2.4$. The track associated with a lepton candidate is required to have both its transverse and longitudinal impact parameters compatible with the position of the PV of the event.

The leptons are required to be isolated; the isolation (*I*) variable is calculated from PF candidates and is corrected for pileup on an event-by-event basis [54]. The scalar $p_{\mathrm {T}}$ sum of all PF candidates reconstructed in an isolation cone with radius $\varDelta R=\sqrt{\smash [b]{(\varDelta \eta )^{2}+(\varDelta \phi )^{2}}}=0.4$ around the lepton’s momentum vector, excluding the lepton itself, is required to be less than 6% of the electron or muon $p_{\mathrm {T}}$ value. For additional offline analysis, the isolated lepton is required to have $p_{\mathrm {T}} >25$ $\,\text {Ge}\text {V}$ for the muon channel and $p_{\mathrm {T}} >30$ $\,\text {Ge}\text {V}$ for the electron channel. Events with more than one lepton satisfying the above requirements are rejected. The lepton flavor samples are exclusive and precedence is given to the selection of muons.

The missing transverse momentum vector, ${\vec p}_{\mathrm {T}} ^{\text {miss}}$, is calculated offline as the negative of the vector sum of transverse momenta of all PF objects identified in the event [55], and the magnitude of this vector is denoted $p_{\mathrm {T}} ^{\text {miss}}$. Events are required to have $p_{\mathrm {T}} ^{\text {miss}}$ in excess of 20$\,\text {Ge}\text {V}$ in the muon channel and 40$\,\text {Ge}\text {V}$ in the electron channel. The tighter requirement for the electron channel reduces the corresponding higher background of QCD multijet events. The transverse mass (${m}_{\mathrm {T}}$) of the lepton and ${\vec p}_{\mathrm {T}} ^{\text {miss}}$ four-vector sum is then required to exceed 40$\,\text {Ge}\text {V}$ in both channels.

Jets are reconstructed by clustering PF candidates with the anti-$k_{\mathrm {T}}$ algorithm [51, 56] using a distance parameter of 0.4. The jet momentum is the vector sum of all particle momenta in the jet and is typically within 5–10% of the true momentum over the whole $p_{\mathrm {T}}$ spectrum and detector acceptance.

An offset correction is applied to jet energies because of the contribution from pileup. Jet energy corrections are derived from simulation, and are confirmed with in situ measurements of the energy balance in dijet, multijet, photon+jet, and Z+jets events with leptonic Z boson decays [57]. Loose jet identification criteria are applied to reject misreconstructed jets resulting from detector noise [58]. Loose criteria are also applied to remove jets heavily contaminated with pileup energy (clustering of energy deposits not associated with a parton from the primary $\mathrm{p} \mathrm{p}$ interaction) [58, 59]. The efficiency of the jet identification is greater than 99%, with a rejection of 90% of background pileup jets with $p_{\mathrm {T}} \simeq 50\,\text {Ge}\text {V}$ and $|\eta |\le 2.5$. For jets with $|\eta | > 2.5$ and $30< p_{\mathrm {T}} < 50\,\text {Ge}\text {V}$, the efficiency is approximately 90% and the pileup jet rejection is approximately 50%. The jet energy resolution (JER) is typically ${\approx }$15% at 10$\,\text {Ge}\text {V}$, 8% at 100$\,\text {Ge}\text {V}$, and 4% at 1$\,\text {Te}\text {V}$ for jets with $|\eta |\le 1$ [57]. Jets reconstructed with $p_{\mathrm {T}} \ge 15$ $\,\text {Ge}\text {V}$ and $|\eta |\le 4.7$ are used in the analysis.

The two highest $p_{\mathrm {T}}$ jets are defined as the tagging jets, and are required to have $p_{\mathrm {T}} >50$ $\,\text {Ge}\text {V}$ and $p_{\mathrm {T}} >30$ $\,\text {Ge}\text {V}$ for the leading and subleading (in $p_{\mathrm {T}}$) jet, respectively. The invariant mass of the two tagging jets is required to satisfy $m_\mathrm {jj}>200\,\text {Ge}\text {V}$.

The transverse momentum of the $\mathrm{W}$ boson ($\vec {p}_{\mathrm {T} \mathrm{W}}$) is evaluated as the vector sum of the lepton $p_{\mathrm {T}}$ and ${\vec p}_{\mathrm {T}} ^{\text {miss}}$. The event $p_{\mathrm {T}}$ balance ($R(p_{\mathrm {T}}$)) is then defined as

1 $$\begin{aligned} R(p_{\mathrm {T}})= \frac{| \vec {p}_{\mathrm {T} \mathrm {j}_1}+\vec {p}_{\mathrm {T} \mathrm {j}_2}+\vec {p}_{\mathrm {T} \mathrm{W}} |}{ |\vec {p}_{\mathrm {T} \mathrm {j}_1} | +|\vec {p}_{\mathrm {T} \mathrm {j}_2} | + |\vec {p}_{\mathrm {T} \mathrm{W}} | } \end{aligned}$$

where $\vec {p}_{\mathrm {T} \mathrm {j}_1}$ and $\vec {p}_{\mathrm {T} \mathrm {j}_2}$ are the transverse momenta of the two tagging jets.

Finally, events are required to have $R(p_{\mathrm {T}}) < 0.2$. This has a negligible effect on the analysis sensitivity and allows the definition of a nonoverlapping control sample with $R(p_{\mathrm {T}}) > 0.2$ that is used to derive a correction to the invariant mass based on a CR in data, as described in Sect. 6.

A multivariate analysis technique, described in Sect. 8, is used to provide an optimal separation of the $\mathrm {DY}\,\mathrm{W} \mathrm {jj}$ and $\mathrm {EW}\,\mathrm{W} \mathrm {jj}$ components of the inclusive $\ell \nu \mathrm {jj}$ spectrum. The main discriminating variables are the dijet invariant mass $m_\mathrm {jj}$ and pseudorapidity separation $\varDelta \eta _\mathrm {jj}$.

Angular variables useful for signal discrimination include the $y^*$ Zeppenfeld variable [6], defined as the difference between the rapidity of the $\mathrm{W}$ boson $y_{\mathrm{W}}$ and the average rapidity of the two tagging jets, i.e.,

2 $$\begin{aligned} y^*=y_{\mathrm{W}}-\frac{1}{2}(y_{\mathrm {j}_1}+y_{\mathrm {j}_2}), \end{aligned}$$

and the $z^*$ Zeppenfeld variable [6] defined as

3 $$\begin{aligned} z^*=\frac{ y^* }{ \varDelta y_\mathrm {jj} }, \end{aligned}$$

where $\varDelta y_\mathrm {jj}$ is the dijet rapidity separation.

Table 1 reports the expected and observed event yields after the initial selection and after imposing a minimum value for the final multivariate discriminant output applied to define the signal-enriched region used for the studies of additional hadronic activity described in Sect. 12.

**Table 1:** Event yields expected for background and signal processes using the initial selections and with a selection on the multivariate analysis output (BDT) that provides similar signal and background yields. The yields are compared to the data observed in the different channels and categories. The total uncertainties quoted for signal, $\mathrm {DY}\,\mathrm{W} \mathrm {jj}$ and diboson backgrounds, and processes with top quarks ($\mathrm{t}{\bar{\mathrm{t}}}$ and single top quarks) include the systematic uncertainties

Sample	Initial		$\mathrm {BDT} > 0.95$	
	$\mu$	$\mathrm{e}$	$\mu$	$\mathrm{e}$
VV	$20,300 \pm 2000$	$9820 \pm 980$	$11.0 \pm 2.5$	$9.6 \pm 2.8$
$\mathrm {DY}\,\mathrm{Z} \mathrm {jj}$	$102,000 \pm 10,000$	$29,900 \pm 3000$	$9.4 \pm 5.9$	$7.7 \pm 3.0$
$\mathrm{t}{\bar{\mathrm{t}}}$	$298,000 \pm 28,000$	$164,000 \pm 15,000$	$146 \pm 17$	$102 \pm 12$
Single top quark	$96,000 \pm 14,000$	$45,800 \pm 6900$	$35.5 \pm 5.6$	$25.7 \pm 4.2$
QCD multijet	$100,000 \pm 39,000$	$65,000 \pm 21,000$	$98 \pm 39$	$17.0 \pm 5.6$
$\mathrm {DY}\,\mathrm{W} \mathrm {jj}$	$1,720,000 \pm 120,000$	$715,000 \pm 51,000$	$356 \pm 65$	$240 \pm 41$
Interference	$7000 \pm 2100$	$3400 \pm 1000$	$18.2 \pm 8.1$	$9.8 \pm 5.5$
Total backgrounds	$2,340,000 \pm 170,000$	$1,032,000 \pm 58,000$	$674 \pm 78$	$412 \pm 44$
$\mathrm {EW}\,\mathrm{W} \mathrm {jj}$ signal	$43,100 \pm 4300$	$20,700 \pm 2100$	$503 \pm 54$	$308 \pm 34$
$\mathrm {EW}\,\mathrm{Z} \mathrm {jj}$ signal	$1330 \pm 130$	$407 \pm 41$	$11.2 \pm 1.3$	$6.6 \pm 0.9$
Total prediction	$2,390,000 \pm 170,000$	$1,054,000 \pm 58,000$	$1186 \pm 95$	$726 \pm 56$
Data	2, 381, 901	1, 051, 285	1138	686

### Discriminating quarks from gluons

Jets in signal events are expected to originate from quarks, whereas for background events it is more probable that jets are initiated by a gluon. A quark-gluon likelihood (QGL) discriminant [11] is evaluated for the two tagging jets with the intent of distinguishing the nature of each jet.

The QGL discriminant exploits differences in the showering and fragmentation of quarks and gluons, making use of the following internal jet composition observables: (1) the particle multiplicity of the jet, (2) the minor root-mean-square of distance between the jet constituents in the $\eta$–$\phi$ plane, and (3) the $p_{\mathrm {T}}$ distribution function of the jet constituents, as defined in Ref. [60].

The variables are used as inputs to a likelihood discriminant on gluon and quark jets constructed from simulated dijet events. The performance of the QGL discriminant is evaluated and validated using independent, exclusive samples of $\mathrm{Z}$+jet and dijet data [60]. Corrections to the simulated QGL distributions and related systematic uncertainties are derived from a comparison of simulation and data distributions.

## The QCD multijet background

The QCD multijet contribution is estimated by defining a multijet-enriched CR with inverted lepton isolation criteria for both the muon and electron channels. In the nominal selection both lepton types are required to pass the relative isolation requirement $I<0.06$, whereas the multijet-enriched CRs are defined with the same event selection but with isolation requirements $0.06<I<0.12$ and $0.06<I<0.15$, for the muon and electron channel respectively. It is then assumed that the $p_{\mathrm {T}} ^{\text {miss}}$ distribution of QCD events has the same shape in both the nominal and the multijet-enriched CR.

The various components, with floating $\mathrm{W}$+jets and QCD multijet background scale factors, are simultaneously fitted to the $p_{\mathrm {T}} ^{\text {miss}}$ data distributions, independently in the muon and electron channels, and the expected QCD multijet yields in the nominal regions are derived.

The contribution of QCD multijet processes in any other observable (*x*) used in the analysis is then normalized to the yields obtained above from the fit to the $p_{\mathrm {T}} ^{\text {miss}}$ distribution, and the shape for the distribution *x* is taken as the difference between data and all simulated background contributions in the *x* distribution in the multijet-enriched CR.

The estimation of the QCD multijet contribution based on a CR in data is validated by checking the modeling of other variables that discriminate QCD multijets from $\mathrm{W}$+jets such as the W transverse mass and the minimum difference in $\phi$ between the missing transerse energy and the jets. Good agreement with the data is observed in all distributions. The stability of the $\mathrm{W}$+jets fitted normalization is checked by varying the selection requirements for the fitted region and repeating the QCD extraction fit. The observed variations in fitted normalization when varying the $m_\mathrm {T}$(W) and $p_{\mathrm {T}} ^{\text {miss}}$ selection requirements with respect to the fit region definition are much smaller than systematic uncertainties.

Although $\mathrm{b}$ tagging is not used in this analysis, a $\mathrm{b}$-tagging discriminant output [61] is used to check the fitted $\mathrm{W}$+jets background normalization as well as the $\mathrm{t}{\bar{\mathrm{t}}}$ normalization from simulation, and they agree with data within the uncertainties. Finally, the selections on $m_\mathrm {jj}$, $p_{\mathrm {T}} ^{\text {miss}}$, and $m_\mathrm {T}$(W) are also loosened in order to verify that the $\mathrm{W}$+jets background scale factor is not biased by these requirements.

## The $m_\mathrm {jj}$ correction

A systematic overestimation of the simulation yields is caused by a partial mistiming of the signals in the forward region of the ECAL endcaps ($2.5<\vert \eta \vert <3.0$). This effect, which increases with increasing $m_\mathrm {jj}$, is observed in both electron and muon channels. A correction for this effect is derived in the nonoverlapping signal-depleted CR obtained by requiring that the event transverse momentum balance $R(p_{\mathrm {T}})$, defined in Sect. 4, exceeds 0.2.

A third-order polynomial correction is first applied to the $\mathrm{W}$+jets simulation separately in the muon and electron channels in order to match the $R(p_{\mathrm {T}})$ distribution in data. The magnitude of the applied $R(p_{\mathrm {T}})$ corrections is about 10%. The uncertainty in this correction due to the limited statistical precision of the simulation as well as data is propagated to the fitted $\mathrm{W}$+jets templates.

A correction to the $m_\mathrm {jj}$ prediction from simulation is derived in the signal-depleted $R(p_{\mathrm {T}})>0.2$ CR via a third-order polynomial fit to the ratio of data to the overall prediction from simulation for signal and background as a function of $\ln (m_\mathrm {jj}/\,\text {Ge}\text {V})$. The electron and muon channels are combined when deriving the $m_\mathrm {jj}$ correction. The uncertainty in the correction includes the data statistical component as well as the systematic uncertainty due to the limited statistical precision of the simulation.

**Fig. 3:**
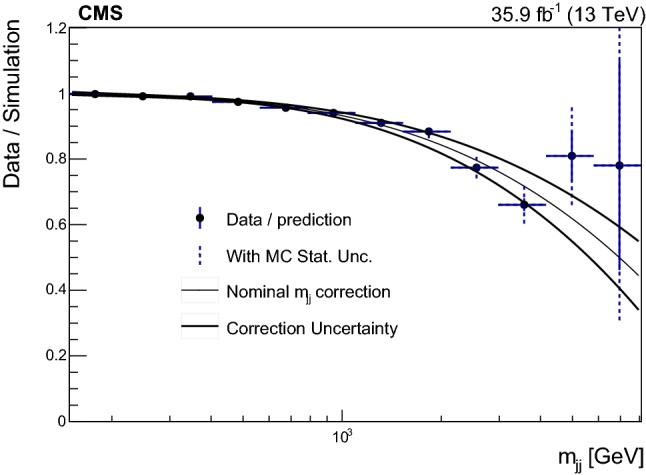
Data divided by simulation as a function of $\ln (m_\mathrm {jj}/\,\text {Ge}\text {V})$ in a signal-depleted control sample with $R(p_{\mathrm {T}})>0.2$. This distribution is fit by a third-order polynomial (solid black line) in order to derive a correction on the simulation $m_\mathrm {jj}$ prediction. The points are varied by the uncertainty, including the effect of the limited number of simulated events and refitted in order to derive the systematic variations on the correction (dashed lines) corresponding to a standard deviation (SD)

Figure 3 shows the fitted correction including the uncertainty. This correction is applied to all simulated results, including the signal, and the corresponding uncertainty is propagated to the signal extraction fits.

## Distributions of discriminating variables

**Fig. 4:**
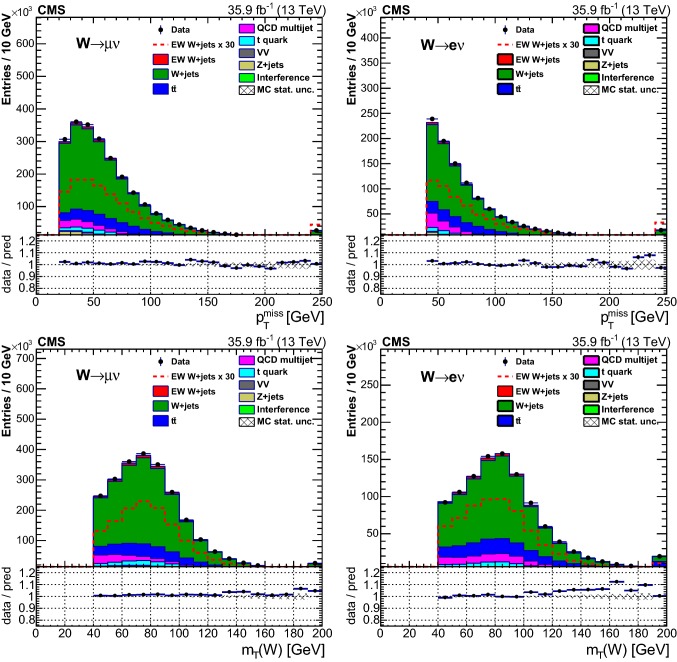
Distribution of the missing transverse momentum (upper) and the lepton–$p_{\mathrm {T}} ^{\text {miss}}$system transverse mass (lower) after the event preselection for the selected leading lepton in the event, in the muon (left) and electron (right) channels. In all plots the last bin contains overflow events

**Fig. 5:**
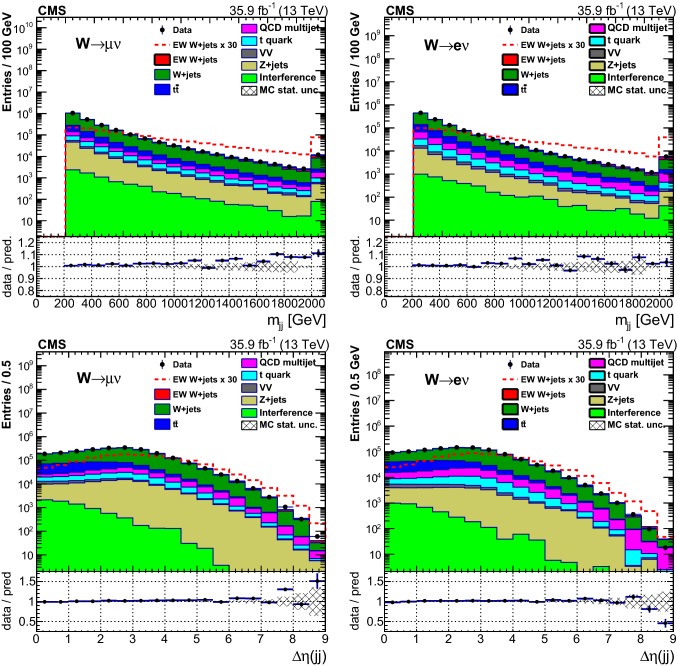
Dijet invariant mass (upper) and pseudorapidity difference (lower) distributions after the event preselection, in the muon (left) and electron (right) channels. In all plots the last bin contains overflow events

**Fig. 6:**
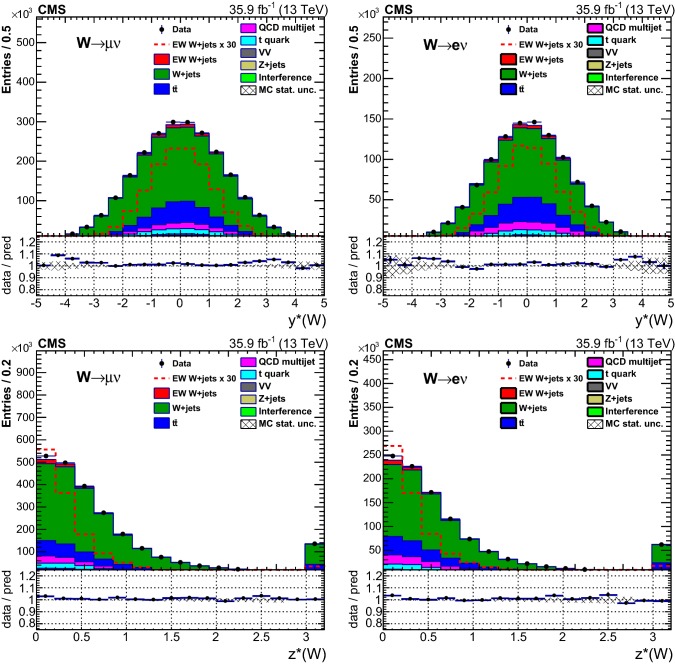
Distributions of the “Zeppenfeld” variables $y^\star$(W) (upper) and $z^\star$(W) (lower) after event preselection in the muon (left) and electron (right) channels. In all plots the first and last bins contain overflow events

**Fig. 7:**
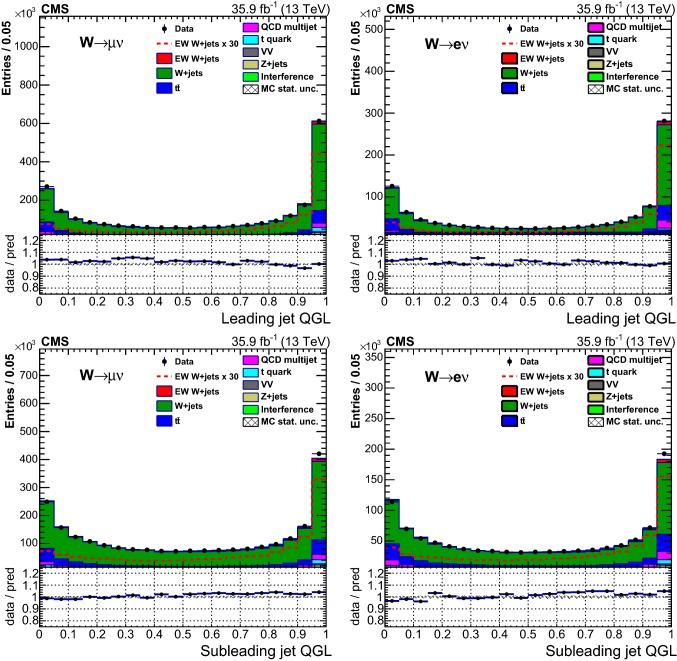
The QGL output for the leading (upper) and subleading (lower) quark jet candidates in the preselected muon (left) and electron (right) samples

Figure 4 shows the $p_{\mathrm {T}} ^{\text {miss}}$ and ${m}_{\mathrm {T}}$(W) distributions after the event preselection. The dijet invariant mass and pseudorapidity difference ($\varDelta \eta _\mathrm {jj}$) after preselection are presented in Fig. 5, and Fig. 6 shows the $y^\star$ and $z^\star$ distributions after the event preselection. The distributions of the QGL likelihood output values in data and simulation for the two tagging jets are shown in Fig. 7. The prediction from simulated events and the data agree within total uncertainties for all discriminating variables.

## Signal discriminants and extraction procedure

The $\mathrm {EW}\,\mathrm{W} \mathrm {jj}$ signal is characterized by a large pseudorapidity separation between the tagging jets, due to the small-angle scattering of the two initial partons. Because of both the topological configuration and the large energy of the outgoing partons, $m_\mathrm {jj}$ is also expected to be large, and can be used to distinguish the $\mathrm {EW}\,\mathrm{W} \mathrm {jj}$ and $\mathrm {DY}\,\mathrm{W} \mathrm {jj}$ processes. The correlation between $\varDelta \eta _\mathrm {jj}$ and $m_\mathrm {jj}$ is expected to be different in signal and background events, therefore these characteristics are expected to yield a high separation power between $\mathrm {EW}\,\mathrm{W} \mathrm {jj}$ and $\mathrm {DY}\,\mathrm{W} \mathrm {jj}$ production. In addition, in signal events it is expected that the $\mathrm{W}$ boson candidate is produced centrally in the rapidity region defined by the two tagging jets. As a consequence, signal events are expected to yield lower values of $z^*$ compared to the DY background. Other variables that are used to enhance the signal-to-background separation are related to the kinematics of the event or to the properties of the jets that are expected to be initiated by quarks. The variables that are used in the multivariate analysis are: (1) $m_\mathrm {jj}$, (2) $\varDelta \eta _\mathrm {jj}$, (3) $z^*$, and (4) the QGL values of the two tagging jets.

The output is built by training a boosted decision tree (BDT) discriminator with the tmva package [62] to achieve an optimal separation between the $\mathrm {EW}\,\mathrm{W} \mathrm {jj}$ and $\mathrm {DY}\,\mathrm{W} \mathrm {jj}$ processes. The simulated events that are used for the BDT training are not used for the signal extraction.

To improve the sensitivity for the extraction of the signal component, the transformation that originally projects the BDT output value in the [−1,$+$1] interval is changed to $\mathrm {BDT'} = \tanh ^{-1}((\mathrm {BDT}+1)/2)$. This allows the purest signal region of the BDT output to be better sampled while keeping an equal-width binning of the BDT variable.

**Fig. 8:**
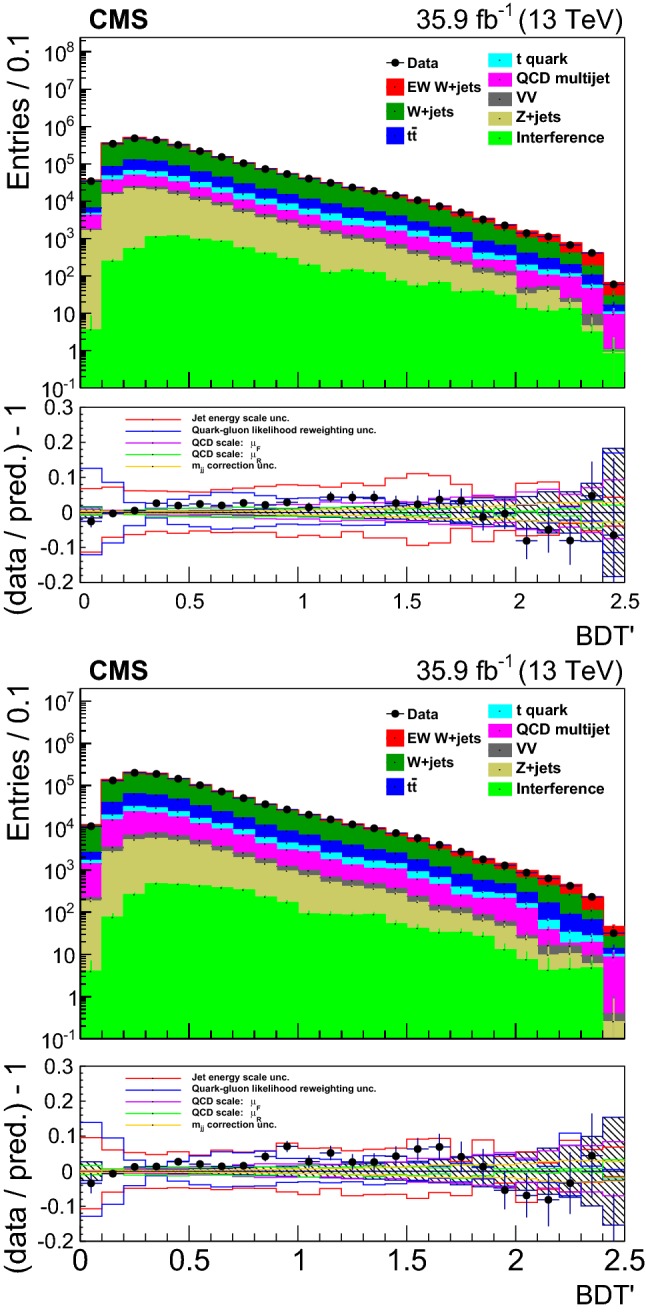
Data and MC simulation BDT’ output distributions for the muon (upper) and electron (lower) channels, using the BDT output transformed with the $\tanh ^{-1}$ function to enhance the purest signal region. The ratio panel shows the statistical uncertainty from the simulation as well as the independent systematic uncertainties front the leading sources

Figure 8 shows the distributions of the discriminants for the two leptonic channels. Good overall agreement between simulation and data is observed in all distributions, and the signal presence is visible at high BDT’ values.

A binned maximum likelihood is built from the expected rates for each process, as a function of the value of the discriminant, which is fit to extract the strength modifiers for the $\mathrm {EW}\,\mathrm{W} \mathrm {jj}$ and $\mathrm {DY}\,\mathrm{W} \mathrm {jj}$ processes, $\mu = \sigma ({\mathrm {EW}~\mathrm{W} \mathrm {jj}}) / \sigma _\mathrm {LO}({\mathrm {EW}~\ell \nu \mathrm {jj}})$ and $\upsilon = \sigma ({\mathrm{W}})/\sigma _\text {NNLO}({\mathrm{W}})$. Nuisance parameters are added to modify the expected rates and shapes according to the estimate of the systematic uncertainties affecting the measurement.

The interference between the $\mathrm {EW}\,\mathrm{W} \mathrm {jj}$ and $\mathrm {DY}\,\mathrm{W} \mathrm {jj}$ processes is included in the fit procedure, and its strength scales as $\sqrt{\mu \upsilon }$. The interference model is derived from the MadGraph 5_amc@nlo simulation described in Sect. 3.

The parameters of the model ($\mu$ and $\upsilon$) are determined by maximizing the likelihood. The statistical methodology follows the one used in other analyses [63] using asymptotic formulas [64]. In this procedure the systematic uncertainties affecting the measurement of the signal and background strengths ($\mu$ and $\upsilon$) are constrained with log-normal probability distributions.

## Systematic uncertainties

The main systematic uncertainties affecting the measurement are classified into experimental and theoretical according to their sources. Some uncertainties affect only normalizations, whereas others affect both the normalization and shape of the BDT output distribution.

### Experimental uncertainties

The following experimental uncertainties are considered.

Integrated luminosity.: A 2.5% uncertainty is assigned to the value of the integrated luminosity [65].
Trigger and selection efficiencies.: Uncertainties in the efficiency corrections based on control samples in data for the leptonic trigger and offline selections are included and amount to a total of 2–3% depending on the lepton $p_{\mathrm {T}}$ and $\eta$, for both the e and $\mu$ channels. These uncertainties are estimated by comparing the lepton efficiencies expected in simulation and measured in data with a “tag-and-probe” method [66].
Jet energy scale and resolution.: The uncertainty in the energy of the jets affects the event selection and the computation of the kinematic variables used to calculate the discriminants. Therefore, the uncertainty in the jet energy scale (JES) affects both the expected event yields and the final shapes. The effect of the JES uncertainty is studied by rescaling up and down the reconstructed jet energy by $p_{\mathrm {T}}$- and $\eta$-dependent scale factors [57]. An analogous approach is used for the JER.
QGL discriminator.: The uncertainty in the performance of the QGL discriminator is measured using independent $\mathrm{Z}$+jet and dijet data, after comparing with the corresponding simulation predictions [60]. Shape variations corresponding to the full differences between the data and the simulation are used as estimates of the uncertainty.
Pileup.: Pileup can affect the identification and isolation of the leptons or the corrected energy of the jets. When the jet clustering algorithm is run, pileup can distort the reconstructed dijet system because of the contamination of tracks and calorimetric deposits. This uncertainty is evaluated by generating alternative distributions of the number of pileup interactions, corresponding to a 4.6% uncertainty in the total inelastic pp cross section at $\sqrt{s}=13\,\text {Te}\text {V}$ [67].
Limited number of simulated events.: For each signal and background simulation, shape variations for the distributions are considered by shifting the content of each bin up or down by its statistical uncertainty [68]. This generates alternatives to the nominal shape that are used to describe the uncertainty from the limited number of simulated events.
$$m_\mathrm {jj} $$ correction.
As described in Sect. 6, the $m_\mathrm {jj}$ prediction from simulation is corrected to match the distribution in data in a signal-depleted $R(p_{\mathrm {T}})>0.2$ control region. The uncertainty in this correction is derived by varying the fitted points within the statistical uncertainty from data and simulation combined and refitting the correction.
QCD multijet background template.: As described in Sect. 5, the QCD multijet prediction is extrapolated from the data in a nonoverlapping CR. The uncertainty in the QCD multijet background template shape is derived by taking the envelope of the shape obtained when varying the lepton isolation requirement used to define the multijet-enriched CR. A 50% uncertainty in the QCD multijet background normalization is also included.

### Theoretical uncertainties

The following theoretical uncertainties are considered in the analysis.

PDF.: The PDF uncertainties are evaluated by comparing the nominal distributions to those obtained when using the alternative PDFs of the NNPDF set, including $\alpha _\mathrm {s}$ variations.
Factorization and renormalization scales.: To account for theoretical uncertainties, signal and background shape variations are built by changing the values of $\mu _\mathrm {F}$ and $\mu _{\mathrm {R}}$ from their defaults by factors of 2 or 1/2 in the ME calculation, simultaneously for $\mu _\mathrm {F}$ and $\mu _{\mathrm {R}}$, but independently for each simulated sample.
Signal acceptance.: A 5% uncertainty on the signal yield is assigned to account for differences between the prediction for the LO signal with respect to the NLO predictions of the vbfnlo generator (v2.6.3).
Normalization of top quark and diboson backgrounds.: Diboson and top quark production processes are modeled with MC simulations. An uncertainty in the normalization of these backgrounds is assigned based on the PDF and $\mu _\mathrm {F}$, $\mu _{\mathrm {R}}$ uncertainties, following calculations in Refs. [42, 43, 47].
Interference between $\mathrm {EW}\,\mathrm{W} \mathrm {jj}$ and $\mathrm {DY}\,\mathrm{W} \mathrm {jj}$.
An overall normalization and a shape uncertainty are assigned to the interference term in the fit, based on an envelope of predictions with different $\mu _\mathrm {F}$, $\mu _{\mathrm {R}}$ scales.
Parton showering model.: The uncertainty in the PS model and the event tune is assessed as the full difference of the acceptance and shape predictions using pythia and herwig++.
$$R(p_{\mathrm {T}})$$ correction.
As described in Sect. 6, the $R(p_{\mathrm {T}})$ prediction from $\mathrm{W}$+jets simulation is corrected to match the distribution in data with all expected contributions other than $\mathrm{W}$+jets subtracted. The uncertainty in this correction is derived by varying the fitted points within the statistical uncertainty from data and simulation combined and refitting the correction.

## Measurement of the $\mathrm {EW}\,\mathrm{W} \mathrm {jj}$ production cross section

**Fig. 9:**
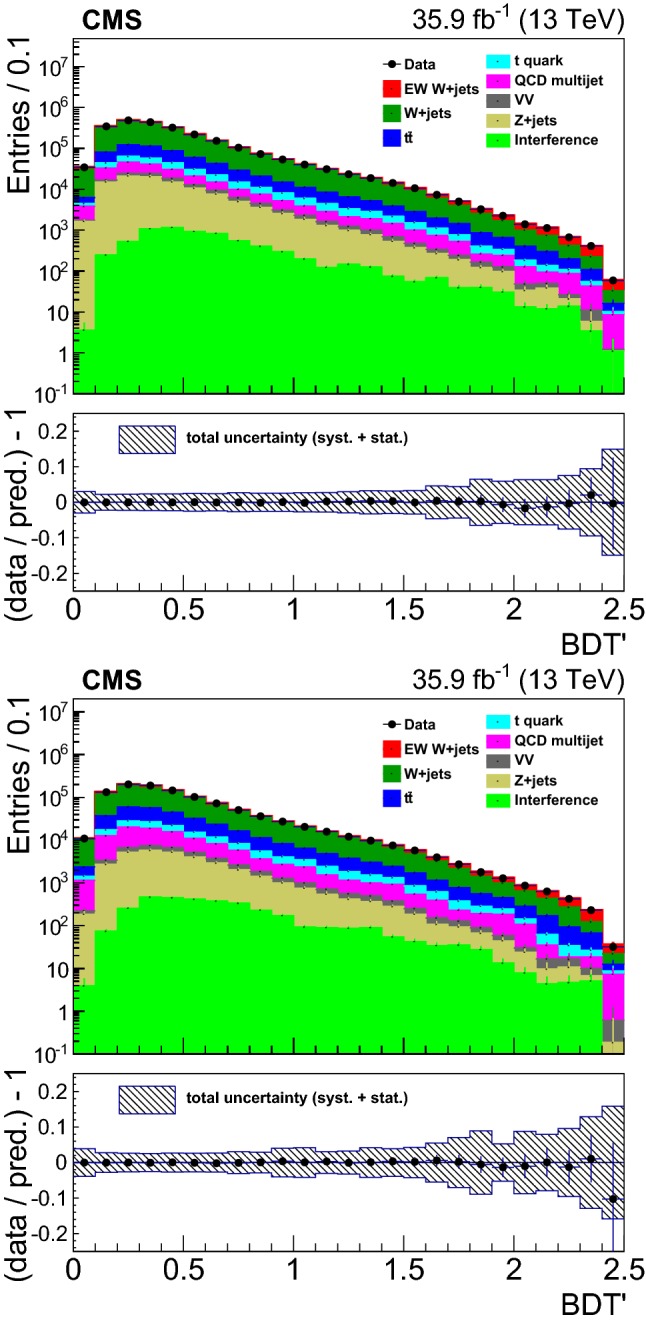
Data compared with simulation for the BDT’ output distribution for the muon (upper) and electron (lower) channels, after the fit. The grey uncertainty band in the ratio panel includes all systematic uncertainties

The signal strength, defined with the $\ell \nu$jj final state in the kinematic region described in Sect. 3, is extracted from the fit to the BDT output distribution as discussed in Sect. 8. Figure 9 shows the BDT distribution in the muon and electron channels for data and simulation after the fit, where the grey uncertainty band includes all systematic uncertainties. Good agreement is observed between the data and simulation within the uncertainties.

In the muon channel, the signal strength is measured to be

$$\begin{aligned} \mu =0.91 \pm 0.02\,\text {(stat)} \pm 0.12\,\text {(syst)} =0.91\pm 0.12\,\text {(total)}, \end{aligned}$$

corresponding to a measured signal cross section

$$\begin{aligned} \begin{aligned} \sigma ({\mathrm {EW}~\ell \nu \mathrm {jj}})&= 6.22 \pm 0.12\,\text {(stat)} \pm 0.74\,\text {(syst)} \,\text {pb} \\&=6.22\pm 0.75 \,\text {(total)}\,\text {pb}. \end{aligned} \end{aligned}$$

In the electron channel, the signal strength is measured to be

$$\begin{aligned} \mu =0.92\pm 0.03\,\text {(stat)} \pm 0.13\,\text {(syst)} =0.92\pm 0.13 \,\text {(total)}, \end{aligned}$$

corresponding to a measured signal cross section

$$\begin{aligned} \begin{aligned} \sigma ({\mathrm {EW}~\ell \nu \mathrm {jj}})&= 6.27 \pm 0.19\,\text {(stat)} \pm 0.80\,\text {(syst)} \,\text {pb} \\&=6.27\pm 0.82\,\text {(total)}\,\text {pb}. \end{aligned} \end{aligned}$$

The results obtained for the different lepton channels are compatible with each other, and in agreement with the SM predictions.

From the combined fit of the two channels, the signal strength is measured to be

$$\begin{aligned} \mu =0.91\pm 0.02\,\text {(stat)} \pm 0.10\,\text {(syst)} =0.91\pm 0.10\,\text {(total)}, \end{aligned}$$

corresponding to a measured signal cross section

$$\begin{aligned} \begin{aligned} \sigma ({\mathrm {EW}~\ell \nu \mathrm {jj}})&= 6.23 \pm 0.12\,\text {(stat)} \pm 0.61\,\text {(syst)} \,\text {pb} \\ {}&=6.23\pm 0.62\,\text {(total)}\,\text {pb}, \end{aligned} \end{aligned}$$

in agreement with the MadGraph 5_amc@nlo LO prediction $\sigma _\mathrm {LO}(\mathrm {EW}\,\ell \nu \mathrm {jj})=6.81^{+0.03}_{-0.06}\,\text {(scale)}\pm 0.26\,\text {(PDF)} \,\text {pb}$. In the combined fit, the DY strength is $\nu =0.88\pm 0.07$. Using the statistical methodology described in Sect. 8, the background-only hypotheses in the electron, muon, and combined channels are all excluded with significance above five standard deviations. Table 2 lists the major sources of uncertainty and their impact on the measured precision of $\mu$. The largest sources of experimental uncertainty are the $m_\mathrm {jj}$ correction, the JES, and the limited number of simulated events, while the largest sources of theoretical uncertainty are the $\mu _\mathrm {F}$, $\mu _{\mathrm {R}}$ scale uncertainties and the uncertainty in the signal acceptance, derived by comparing the LO signal prediction with the prediction from the vbfnlo generator.

**Table 2:** Major sources of uncertainty in the measurement of the signal strength $\mu$, and their impact. The total uncertainty is separated into four components: statistical, number of simulated events, experimental, and theory. The experimental and theory components are further decomposed into their primary individual uncertainty sources

Uncertainty source	$\varDelta \mu$	
Statistical	$+0.02$	$-0.02$
Size of simulated samples	$+0.05$	$-0.05$
Experimental	$+0.07$	$-0.07$
Jet energy scale and resolution	$+0.03$	$-0.01$
QCD multijet estimation	$+0.03$	$-0.03$
$m_{jj}$ correction	$+0.05$	$-0.05$
Background normalization	$+0.02$	$-0.02$
Other experimental uncertainties	$<0.01$	
Theory	$+0.07$	$-0.07$
QCD scale and PDF	$+0.05$	$-0.05$
Interference	$+0.02$	$-0.02$
Signal acceptance	$+0.05$	$-0.05$
Other theory uncertainties	$+0.01$	$-0.01$
Total	$+0.10$	$-0.10$

The signal strength is also measured with respect to the NLO signal prediction, as described in Sect. 3. In the muon channel, the signal strength is measured to be

$$\begin{aligned} \mu _{\mathrm {NLO}}=0.91 \pm 0.02\,\text {(stat)} \pm 0.12\,\text {(syst)} =0.91\pm 0.12\,\text {(total)}. \end{aligned}$$

In the electron channel, the signal strength is measured to be

$$\begin{aligned} \mu _{\mathrm {NLO}}=0.89 \pm 0.03\,\text {(stat)} \pm 0.12\,\text {(syst)} =0.89\pm 0.12\,\text {(total)}. \end{aligned}$$

From the combined fit of the two channels, the signal strength is measured to be

$$\begin{aligned} \mu _{\mathrm {NLO}}=0.90 \pm 0.02\,\text {(stat)} \pm 0.10\,\text {(syst)} =0.90\pm 0.10\,\text {(total)}, \end{aligned}$$

corresponding to a measured signal cross section

$$\begin{aligned} \begin{aligned} \sigma ({\mathrm {EW}~\ell \nu \mathrm {jj}})&= 6.07 \pm 0.12\,\text {(stat)} \pm 0.57\,\text {(syst)} \,\text {pb} \\ {}&=6.07\pm 0.58\,\text {(total)}\,\text {pb}, \end{aligned} \end{aligned}$$

in agreement with the powheg NLO prediction $\sigma _\mathrm {NLO}(\mathrm {EW}\,\ell \nu \mathrm {jj})=6.74^{+0.02}_{-0.04}\,\text {(scale)}\pm 0.26\,\text {(PDF)} \,\text {pb}$.

## Limits on anomalous gauge couplings

It is useful to look for signs of new physics via a model-independent EFT framework. In the framework of EFT, new physics can be described as an infinite series of new interaction terms organized as an expansion in the mass dimension of the operators.

In the EW sector of the SM, the first higher-dimensional operators containing bosons are six-dimensional [8]:

4 $$\begin{aligned} \begin{aligned} {\mathcal {O}}_{WWW}&= \frac{c_{WWW}}{\varLambda ^2}W_{\mu \nu }W^{\nu \rho }W_{\rho }^{\mu },\\ {\mathcal {O}}_{W}&= \frac{c_{W}}{\varLambda ^2}(D^{\mu }\varPhi )^{\dagger }W_{\mu \nu }(D^{\nu }\varPhi ),\\ {\mathcal {O}}_{B}&= \frac{c_{B}}{\varLambda ^2}(D^{\mu }\varPhi )^{\dagger }B_{\mu \nu }(D^{\nu }\varPhi ),\\ \widetilde{{\mathcal {O}}}_{WWW}&= \frac{{\widetilde{c}}_{WWW}}{\varLambda ^2}{\widetilde{W}}_{\mu \nu }W^{\nu \rho }W_{\rho }^{\mu },\\ \widetilde{{\mathcal {O}}}_{W}&= \frac{{\widetilde{c}}_{W}}{\varLambda ^2}(D^{\mu }\varPhi )^{\dagger }{\widetilde{W}}_{\mu \nu }(D^{\nu }\varPhi ), \end{aligned} \end{aligned}$$

where, as is customary, group indices are suppressed and the mass scale $\varLambda$ is factorized from the coupling constants *c*. In Eq. (4), $W_{\mu \nu }$ is the SU(2) field strength, $B_{\mu \nu }$ is the U(1) field strength, $\varPhi$ is the Higgs doublet, and operators with a tilde are the magnetic duals of the field strengths. The first three operators are charge and parity conserving, whereas the last two are not. Models with operators that preserve charge conjugation and parity symmetries can be included in the calculation either individually or in pairs. With these assumptions, the values of coupling constants divided by the mass scale $c/\varLambda ^2$ are measured.

These operators have a rich phenomenology since they contribute to many multiboson scattering processes at tree level. The operator ${\mathcal {O}}_{WWW}$ modifies vertices with three or six vector bosons, whereas the operators ${\mathcal {O}}_{W}$ and ${\mathcal {O}}_{B}$ modify both the HVV vertices and vertices with three or four vector bosons. A more detailed description of the phenomenology of these operators can be found in Ref. [69]. Modifications to the ZWW and $\gamma$WW vertices are investigated in this analysis, since these modify the $\mathrm{p} \mathrm{p} \rightarrow \mathrm{W} \mathrm {jj}$ cross section.

Previously, modifications to these vertices have been studied using anomalous trilinear gauge couplings [70]. The relationship between the dimension-six operators in Eq. (4) and ATGCs can be found in Ref. [8]. Most stringent limits on ATGC parameters were previously set by LEP [71], CDF [72], D0 [73], ATLAS [74, 75], and CMS [76, 77].

### Statistical analysis

The measurement of the coupling constants uses templates in the $p_{\mathrm {T}}$ of the lepton from the $W\rightarrow \ell \nu$ decay. Because this is well measured and longitudinally Lorentz invariant, this variable is robust against mismodeling and ideal for this purpose. An additional requirement of $\mathrm {BDT} >0.5$ has been applied, which is optimized based on the expected sensitivity to the ATGC signal. The expected limits are subsequently improved by 20–25% with respect to the expected limits without a BDT selection. In each channel, four bins from $0< p_{\mathrm {T}} ^\ell < 1.2 \,\text {Te}\text {V}$ are used, where the last bin contains overflow and its lower bin edge boundary has been optimized separately for each channel.

For each signal MC event, 125 weights are assigned that correspond to a $5{\times } 5{\times } 5$ grid in $(c_{WWW}/\varLambda ^2) \, (c_{W}/\varLambda ^2) \, (c_{B}/\varLambda ^2)$. Equal bins are used in the interval $[-15, 15]\,\text {Te}\text {V} ^{-2}$ for $c_{WWW}/\varLambda ^2$, $[-40, 40]\,\text {Te}\text {V} ^{-2}$ for $c_{W}/\varLambda ^2$, and equal bins in the interval $[-175, 175]\,\text {Te}\text {V} ^{-2}$ for $c_{B}/\varLambda ^2$.

**Fig. 10:**
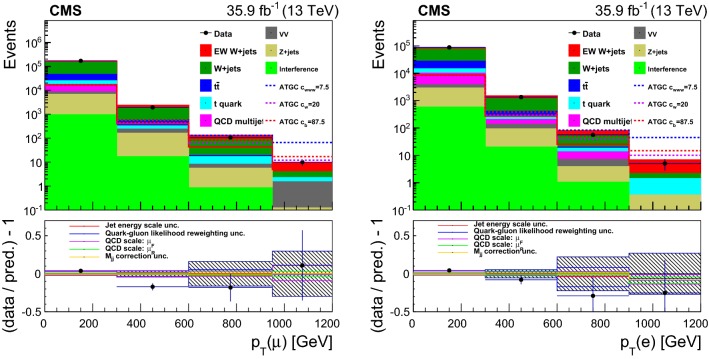
Distributions of $p_{\mathrm {T}} ^\ell$ in data and SM backgrounds, and various ATGC scenarios in the muon (left) and electron (right) channels, before the fit. For each ATGC scenario plotted a particular parameter is varied while the other ATGC parameters are fixed to zero. The lower panels show the ratio between data and prediction minus one with the statistical uncertainty from simulation (grey hatched band) as well as the leading systematic uncertainties in the shape of the $p_{\mathrm {T}} ^\ell$ distribution

To construct the $p_{\mathrm {T}} ^\ell$ templates, the associated weights calculated for each event are used to construct a parametrized model of the expected yield in each bin as a function of the values of the dimension-six operators’ coupling constants. For each bin, the ratios of the expected signal yield with dimension-six operators to the one without (leaving only the SM contribution) are fitted at each point of the grid to a quadratic polynomial. The highest $p_{\mathrm {T}} ^\ell$ bin has the largest statistical power to detect the presence of higher-dimensional operators. Figure 10 shows examples of the final templates, with the expected signal overlaid on the background expectation, for three different hypotheses of dimension-six operators. The SM distribution is normalized to the expected cross section.

A simultaneous binned fit for the values of the ATGCs is performed in the two lepton channels. A profile likelihood method, the Wald Gaussian approximation, and Wilks’ theorem [78] are used to derive confidence intervals at 95% confidence level (CL). One-dimensional and two-dimensional limits are derived on each of the three ATGC parameters and each combination of two ATGC parameters while all other parameters are set to their SM values. Systematic and theoretical uncertainties are represented by the individual nuisance parameters with log-normal distributions and are profiled in the fit.

### Results

No significant deviation from the SM expectation is observed. Limits on the EFT parameters are reported and also translated into the equivalent parameters defined in an effective Lagrangian (LEP parametrization) in Ref. [79], without form factors: $\lambda ^{\gamma } = \lambda ^{\mathrm{Z}} = \lambda$, $\varDelta {\kappa ^{\mathrm{Z}}} = \varDelta {g_1^{\mathrm{Z}}}-\varDelta {\kappa ^\gamma } \, \tan ^2\theta _{{\mathrm{W}}}$. The parameters $\lambda$, $\varDelta {\kappa ^{Z}}$, and $\varDelta {g_1^{\mathrm{Z}}}$ are considered, where the $\varDelta$ symbols represent deviations from their respective SM values.

**Fig. 11:**
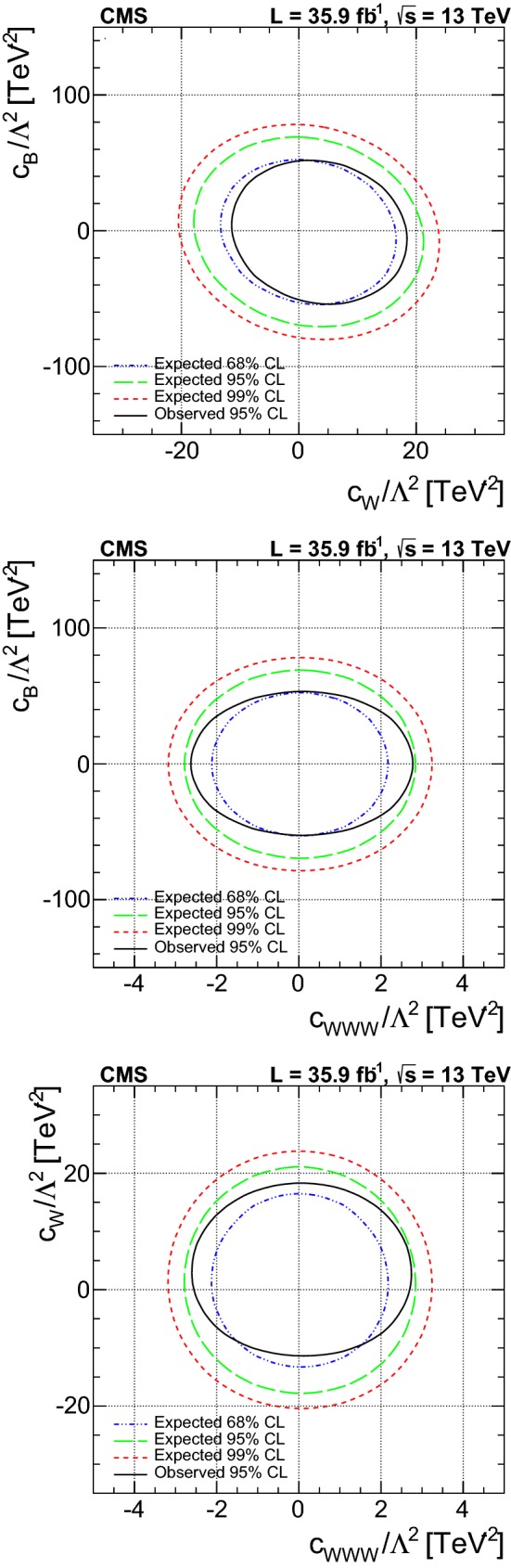
Expected and observed two-dimensional limits on the EFT parameters at 95% CL

**Fig. 12:**
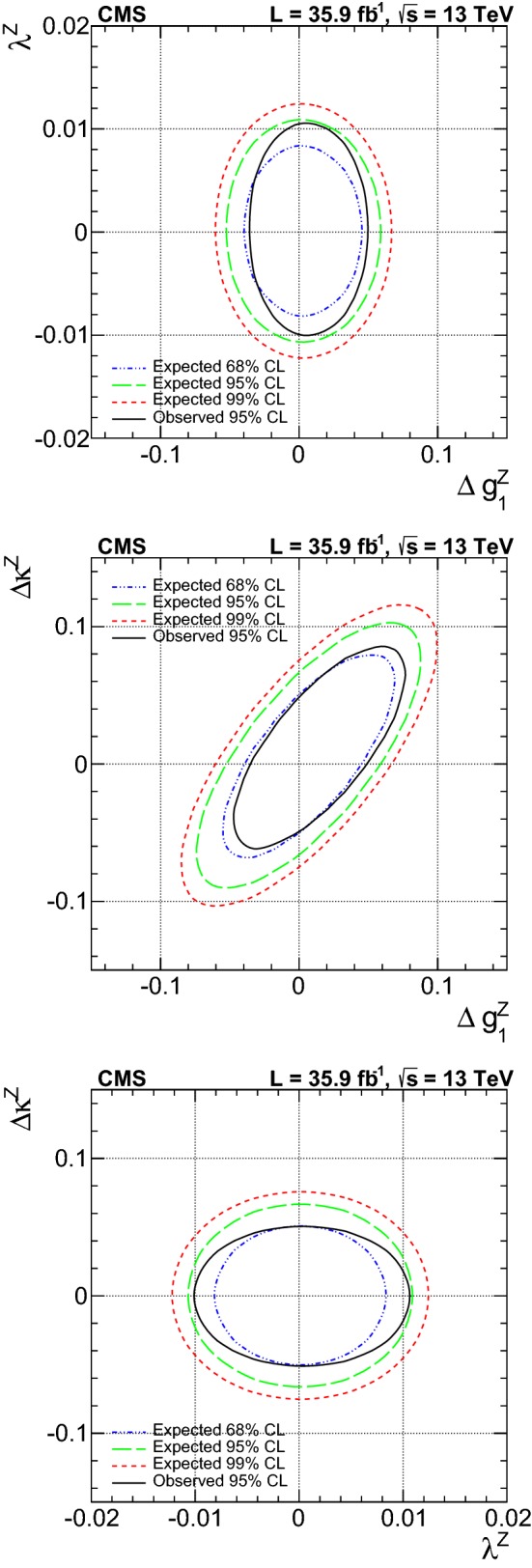
Expected and observed two-dimensional limits on the ATGC effective Lagrangian (LEP parametrization) parameters at 95% CL

Results for the one-dimensional limits are listed in Table 3 for $c_{WWW}$, $c_W$ and $c_B$, and in Table 4 for $\lambda$, $\varDelta g_{1}^{\mathrm{Z}}$ and $\varDelta \kappa _{1}^{\mathrm{Z}}$; two-dimensions limits are shown in Figs. 11 and  12. The results are dominated by the sensitivity in the muon channel due to the larger acceptance for muons. An ATGC signal is not included in the interference between EW and DY production. The effect on the limits is small (${<}$3%). The LHC semileptonic WZ analysis using 13$\,\text {Te}\text {V}$ data currently sets the most stringent limits on $c_{WWW}/\varLambda ^2$ and $c_W/\varLambda ^2$, while the WW analysis using 8$\,\text {Te}\text {V}$ data currently sets the tightest limits on $c_B/\varLambda ^2$. This analysis is most sensitive to $c_{WWW}/\varLambda ^2$, where the limit is slightly less restrictive but comparable.

**Table 3:** One-dimensional limits on the ATGC EFT parameters at 95% CL

Coupling constant	Expected 95% CL interval ($\text {Te}\text {V} {}^{-2}$)	Observed 95% CL interval ($\text {Te}\text {V} {}^{-2}$)
$c_{WWW}/\varLambda ^{2}$	$[-2.5, 2.5]$	$[-2.3, 2.5]$
$c_{W}/\varLambda ^{2}$	$[-16, 19]$	$[-8.8, 16]$
$c_{B}/\varLambda ^{2}$	$[-62, 61]$	$[-45,46]$

**Table 4:** One-dimensional limits on the ATGC effective Lagrangian (LEP parametrization) parameters at 95% CL

Coupling constant	Expected 95% CL interval	Observed 95% CL interval
$\lambda ^{Z}$	$[-0.0094, 0.0097]$	$[-0.0088, 0.0095]$
$\varDelta g_{1}^{Z}$	$[-0.046, 0.053]$	$[-0.029, 0.044]$
$\varDelta \kappa _{1}^{Z}$	$[-0.059, 0.059]$	$[-0.044, 0.044]$

### Combination with the VBF Z boson production analysis

**Fig. 13:**
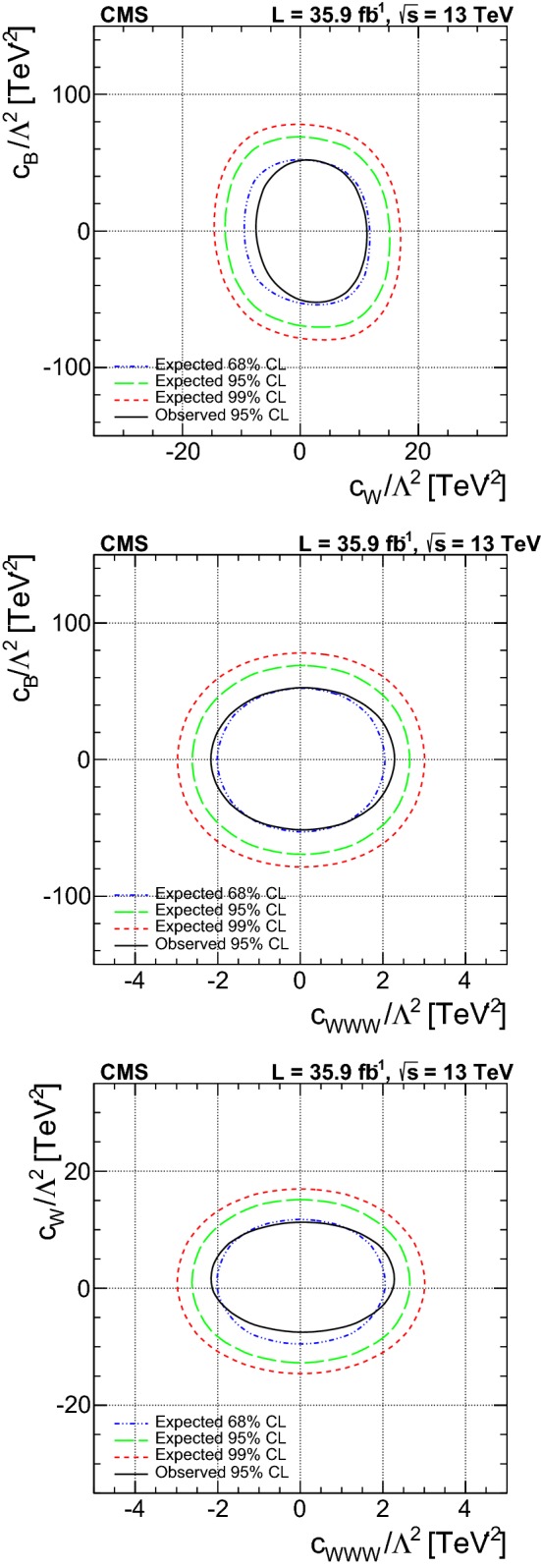
Expected and observed two-dimensional limits on the EFT parameters at 95% CL from the combination of EW $\mathrm{W} \mathrm {jj}$ and EW $\mathrm{Zjj}$ analyses

**Fig. 14:**
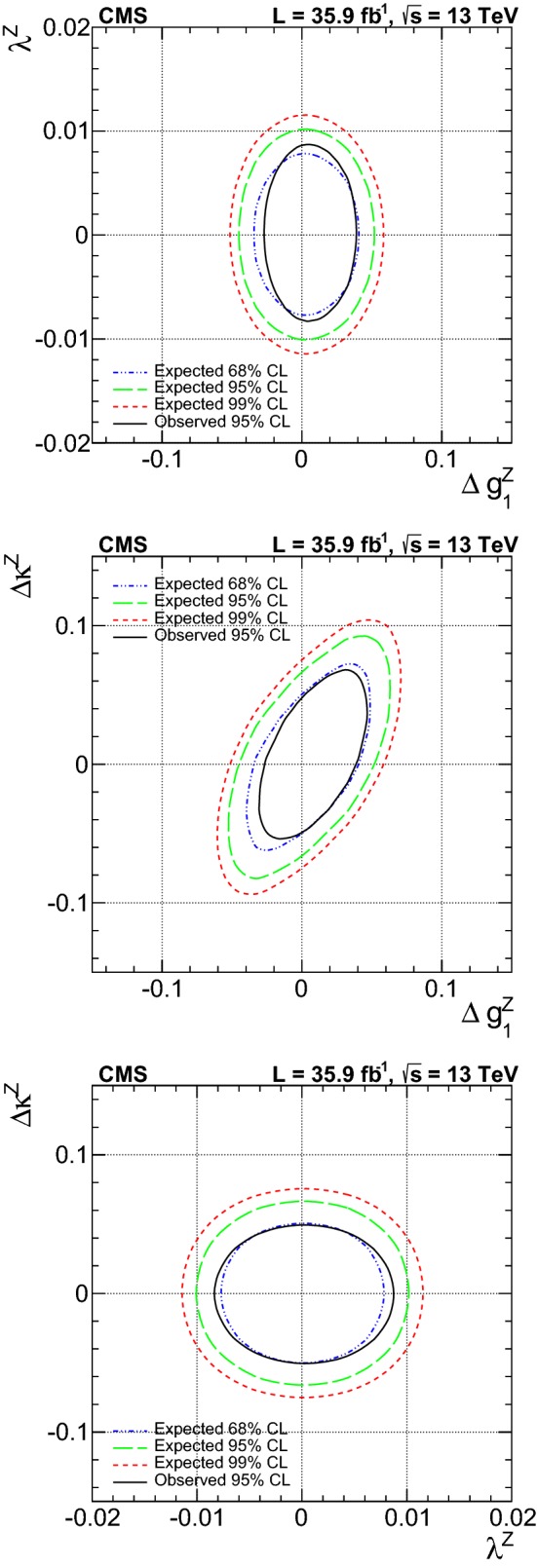
Expected and observed two-dimensional limits on the ATGC effective Lagrangian (LEP parametrization) parameters at 95% CL from the combination of EW $\mathrm{W} \mathrm {jj}$ and EW $\mathrm{Zjj}$ analyses

As mentioned in Sect. 1, the closely-related EW $\mathrm{Zjj}$ process has been measured by CMS at $\sqrt{s}=13\,\text {Te}\text {V}$ [15]. This result included constraints on ATGC EFT parameters obtained via a fit to the $p_{\mathrm {T}}$(Z) distribution, an experimentally clean observable sensitive to deviations from zero in the ATGC parameters. Both the EW $\mathrm{Zjj}$ and EW $\mathrm{W} \mathrm {jj}$ analyses are sensitive to anomalous couplings related to the WWZ vertex. A simultaneous binned likelihood fit for the ATGC parameters is performed to the $p_{\mathrm {T}}$(Z) distribution in the EW Zjj production and and $p_{\mathrm {T}} ^\ell$ in the EW $\mathrm{W} \mathrm {jj}$ production. In the combined fit, the primary uncertainty sources are correlated including the JES and JER uncertainties. Results for the one-dimensional limits are listed in Table 5 for $c_{WWW}$, $c_W$ and $c_B$, and in Table 6 for $\lambda$, $\varDelta g_{1}^{\mathrm{Z}}$, and $\varDelta \kappa _{1}^{\mathrm{Z}}$; two-dimensions limits are shown in Figs. 13 and  14.

**Table 5:** One-dimensional limits on the ATGC EFT parameters at 95% CL from the combination of EW $\mathrm{W} \mathrm {jj}$ and EW $\mathrm{Zjj}$ analyses

Coupling constant	Expected 95% CL interval ($\text {Te}\text {V} {}^{-2}$)	Observed 95% CL interval ($\text {Te}\text {V} {}^{-2}$)
$c_{WWW}/\varLambda ^{2}$	$[-2.3, 2.4]$	$[-1.8, 2.0]$
$c_{W}/\varLambda ^{2}$	$[-11, 14]$	$[-5.8, 10.0]$
$c_{B}/\varLambda ^{2}$	$[-61, 61]$	$[-43,45]$

**Table 6:** One-dimensional limits on the ATGC effective Lagrangian (LEP parametrization) parameters at 95% CL from the combination of EW $\mathrm{W} \mathrm {jj}$ and EW $\mathrm{Zjj}$ analyses

Coupling constant	Expected 95% CL interval	Observed 95% CL interval
$\lambda ^{Z}$	$[-0.0089, 0.0091]$	$[-0.0071, 0.0076]$
$\varDelta g_{1}^{Z}$	$[-0.040, 0.047]$	$[-0.021, 0.034]$
$\varDelta \kappa _{1}^{Z}$	$[-0.058, 0.059]$	$[-0.043, 0.042]$

## Study of the hadronic and jet activity in $\mathrm{W}$+jet events

Having established the presence of the SM signal, the properties of the hadronic activity in the selected events can be examined, in particular in the the region in rapidity between the two tagging jets, with low expected hadron activity (rapidity gap). The production of additional jets in the rapidity gap, in a region with a larger contribution of $\mathrm {EW}\,\mathrm{W} \mathrm {jj}$ processes is explored in Sect. 12.1. Studies of the rapidity gap hadronic activity using track-only observables, are presented in Sect. 12.2. Finally, a study of hadronic activity vetoes, using both PF jets and track-only observables, is presented in Sect. 12.3. A significant suppression of the hadronic activity in signal events is expected because the final-state objects originate from EW interactions, in contrast with the radiative QCD production of jets in $\mathrm {DY}\,\mathrm{W} \mathrm {jj}$ events.

In all these studies, event distributions are shown with a selection on the output value at BDT $>0.95$, which allows a signal-enriched region to be selected with a similar fraction of signal and background events. None of the BDT input observables listed in Sect. 8 are related to additional hadronic activity observables, as a consequence there is no bias on the additional hadronic activity observables due to the BDT output cut. The reconstructed distributions are compared directly to the prediction obtained with a full simulation of the CMS detector. In the BDT $>0.95$ region, the dominant uncertainty on the prediction from simulation is due to the limited number of generated events.

### Jet activity studies in a high-purity region

For this study, aside from the two tagging jets used in the preselection, all PF jets with $p_{\mathrm {T}} >15\,\text {Ge}\text {V}$ found within the pseudorapidity gap of the tagging jets, $\eta ^\text {tag jet}_\text {min}< \eta < \eta ^\text {tag jet}_\text {max}$, are used. For the estimation of the background contributions, the normalizations obtained from the fit discussed in Sect. 10 are used.

**Fig. 15:**
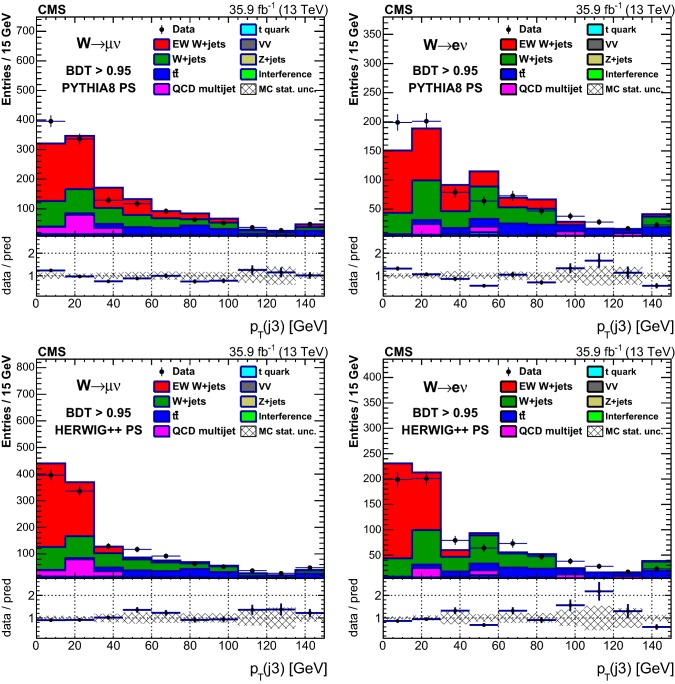
Leading additional jet $p_{\mathrm {T}}$ ($p_{\mathrm {T}}$ (j3)) for $\mathrm {BDT} > 0.95$ in the muon (left) and electron (right) channels including the signal prediction from MadGraph 5_amc@nlo interfaced with pythia parton showering (upper) and herwig++ parton showering (lower). In all plots the last bin contains overflow events, and the first bin contains events where no additional jet with $p_{\mathrm {T}}$ $>15$ $\,\text {Ge}\text {V}$ is present

**Fig. 16:**
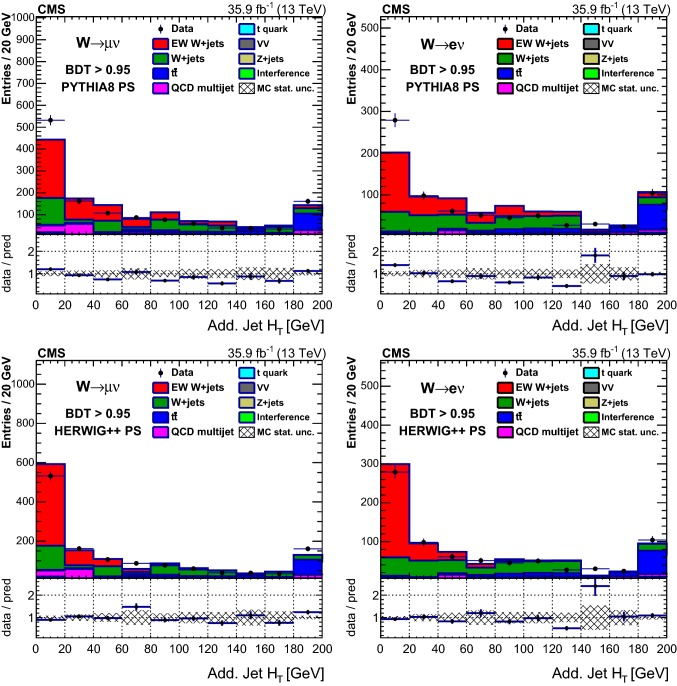
Total $H_{\mathrm {T}}$ of the additional jets for $\mathrm {BDT} > 0.95$ in the muon (left) and electron (right) channels including the signal prediction from MadGraph 5_amc@nlo interfaced with pythia parton showering (upper) and herwig++ parton showering (lower). In all plots the last bin contains overflow events, and the first bin contains events where no additional jet with $p_{\mathrm {T}}$ $>15$ $\,\text {Ge}\text {V}$ is present

The $p_{\mathrm {T}}$ of the leading additional jet in $\mathrm{W} \mathrm {jj}$ events, as well as the scalar $p_{\mathrm {T}}$ sum ($H_{\mathrm {T}}$) of all additional jets, are shown in Figs. 15 and 16, comparing data and simulations including the signal prediction from MadGraph 5_amc@nlo interfaced with either pythia or herwig++ parton showering. The comparison reveals a deficit in the simulation predictions with pythia parton showering for the rate of events with lower additional jet activity, whereas the tail of higher additional activity is generally in better agreement.

A suppression of additional jets is observed in data compared with the background-only simulation shapes. In the simulation of the signal, the additional jets are produced by the PS (see Sect. 3), so studying these distributions provides insight on the PS model in the rapidity gap region.

### Study of charged hadron activity

For this study, a collection is formed of high-purity tracks [80] with $p_{\mathrm {T}} > 0.3\,\text {Ge}\text {V}$, uniquely associated with the main PV in the event. Tracks associated with the lepton or with the tagging jets are excluded from the selection. The association between the selected tracks and the reconstructed PVs is carried out by minimizing the longitudinal impact parameter, which is defined as the *z*-distance between the PV and the point of closest approach of the track helix to the PV, labeled $d_z^\mathrm {PV}$. The association is required to satisfy the conditions $d_z^\mathrm {PV}<2\,\text {mm}$ and $d_z^\mathrm {PV}<3\delta d_z^\mathrm {PV}$, where $\delta d_z^\mathrm {PV}$ is the uncertainty in $d_z^\mathrm {PV}$.

A collection of “soft-track” jets is defined by clustering the selected tracks using the anti-$k_{\mathrm {T}}$ clustering algorithm [51] with a distance parameter of $R=0.4$. The use of track jets represents a clean and well-understood method [81] to reconstruct jets with energy as low as a few $\,\text {Ge}\text {V}$. These jets are not affected by pileup because of the association of the constituent tracks with the hard scattering vertex [82].

**Fig. 17:**
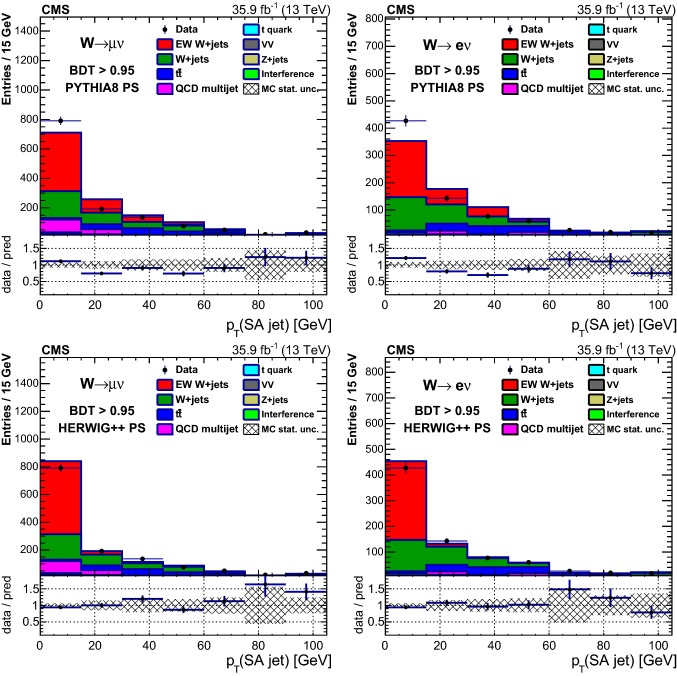
Leading additional soft-activity (SA) jet $p_{\mathrm {T}}$ for BDT $> 0.95$ in the muon (left) and electron (right) channels including the signal prediction from MadGraph 5_amc@nlo interfaced with pythia parton showering (upper) and herwig++ parton showering (lower)

**Fig. 18:**
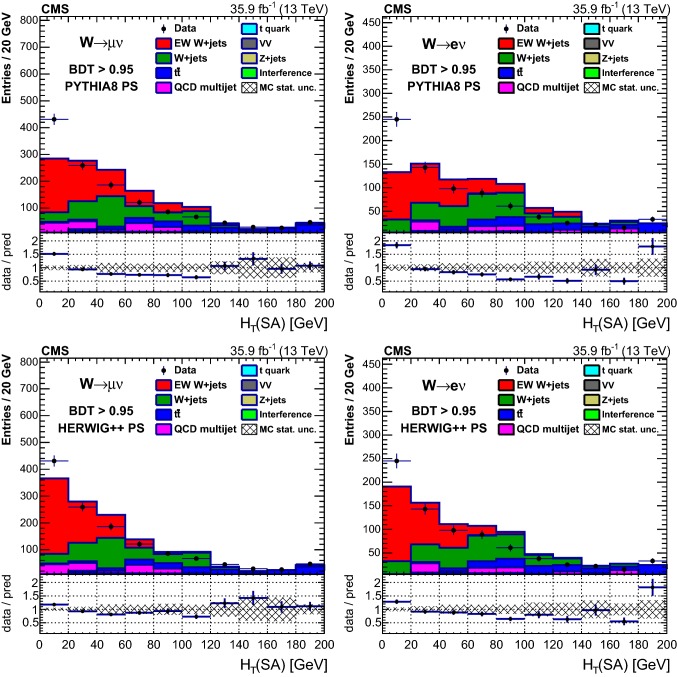
Total soft activity (SA) jet $H_{\mathrm {T}}$ for BDT $> 0.95$ in the muon (left) and electron (right) channels including the signal prediction from MadGraph 5_amc@nlo interfaced with pythia parton showering (upper) and herwig++ parton showering (lower). In all plots the last bin contains overflow events

Track jets of low $p_{\mathrm {T}}$ and within $\eta ^\text {tag jet}_\text {min}< \eta < \eta ^\text {tag jet}_\text {max}$ are considered for the study of the hadronic activity between the tagging jets, and referred to as “soft activity” (SA). For each event, the scalar $p_{\mathrm {T}}$ sum of the soft-track jets with $p_{\mathrm {T}}$ $>1$ $\,\text {Ge}\text {V}$ is computed, and referred to as the “soft $H_{\mathrm {T}}$” variable. Figures 17 and 18 show the distribution of the leading soft-track jet $p_{\mathrm {T}}$ and soft $H_{\mathrm {T}}$ in the signal-enriched region (BDT $>0.95$), for the electron and muon channels, compared to predictions from pythia and herwig++ PS models. The plots show some disagreement between the data and the predictions, in particular in the regions of small additional activity, when compared with the pythia predictions.

### Study of hadronic activity vetoes

The efficiency of a hadronic activity veto corresponds to the fraction of events with a measured gap activity below a given threshold. This efficiency is studied as a function of the applied threshold for various gap activity observables. The veto thresholds studied here start at 15$\,\text {Ge}\text {V}$ for gap activities measured with standard PF jets, while they go down to 1$\,\text {Ge}\text {V}$ for gap activities measured with soft-track jets.

**Fig. 19:**
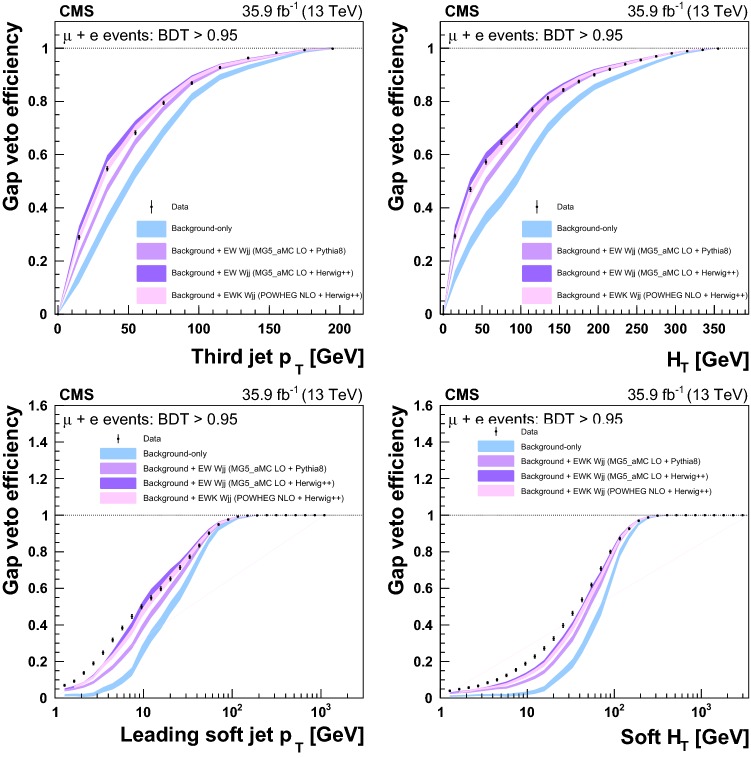
Hadronic activity veto efficiencies in the signal-enriched $\mathrm {BDT}>0.95$ region for the muon and electron channels combined, as a function of the leading additional jet $p_{\mathrm {T}}$ (upper left), additional jet $H_{\mathrm {T}}$ (upper right), leading soft-activity jet $p_{\mathrm {T}}$ (lower left), and soft-activity jet $H_{\mathrm {T}}$ (lower right). The data are compared with the background-only prediction as well as background+signal with pythia parton showering and background+signal with herwig++ parton showering. In addition, the background+signal prediction from powheg plus herwig++ parton showering is included. The uncertainty bands include only the statistical uncertainty in the prediction from simulation, and the data points include only the statistical uncertainty in data

Figure 19 shows the gap activity veto efficiency of combined muon and electron events in the signal-enriched region when placing an upper threshold on the $p_{\mathrm {T}}$ of the additional third jet, on the $H_{\mathrm {T}}$ of all additional jets, on the leading soft-activity jet $p_{\mathrm {T}}$, or on the soft-activity $H_{\mathrm {T}}$. The observed efficiency in data is compared to expected efficiencies for background-only events, and efficiencies for background plus signal events where the signal is modeled with pythia or herwig++. Data points clearly disfavor the background-only predictions and are in reasonable agreement with the presence of the signal with the herwig++ PS predictions for gap activities above 20$\,\text {Ge}\text {V}$, while the signal with pythia PS seems to generally overestimate the gap activity. In the events with very low gap activity, in particular below 10$\,\text {Ge}\text {V}$ as measured with the soft track jets, the data indicates gap activities also below the herwig++ PS predictions. In addition, the expected efficiencies are included for background plus signal events where the signal is modeled with powheg (Sect. 3) with herwig++ PS. The powheg plus herwig++ prediction is in good agreement with the LO plus herwig++ prediction.

## Summary

The cross section of the electroweak production of a $\mathrm{W}$ boson in association with two jets is measured in the kinematic region defined as invariant mass $m_\mathrm {jj} >120\,\text {Ge}\text {V}$ and transverse momenta $p_\mathrm {T j} > 25\,\text {Ge}\text {V}$. The data sample corresponds to an integrated luminosity of $35.9~\mathrm {fb}^{-1}$ of proton–proton collisions at centre-of-mass energy $\sqrt{s}=13\,\text {Te}\text {V}$ recorded by the CMS Collaboration at the LHC. The measured cross section $\sigma _\mathrm {EW}(\mathrm{W} \mathrm {jj})= 6.23 \pm 0.12 \,\text {(stat)} \pm 0.61 \,\text {(syst)} \,\text {pb}$ agrees with the leading order standard model prediction. This is the first observation of this process at $\sqrt{s}=13\,\text {Te}\text {V}$.

A search is performed for anomalous trilinear gauge couplings associated with dimension-six operators as given in the framework of an effective field theory. No evidence for ATGCs is found, and the corresponding 95% confidence level intervals on the dimension-six operators are $-2.3< c_{{\mathrm{W} \mathrm{W} \mathrm{W}}}/\varLambda ^2 < 2.5\,\text {Te}\text {V} ^{-2}$, $-8.8< c_{\mathrm{W}}/\varLambda ^2 < 16\,\text {Te}\text {V} ^{-2}$, and $-45< c_{\mathrm {B}}/\varLambda ^2 < 46\,\text {Te}\text {V} ^{-2}$. These results are combined with previous results on the electroweak production of a Z boson in association with two jets, yielding the limit on the $c_{{\mathrm{W} \mathrm{W} \mathrm{W}}}$ coupling $-1.8< c_{{\mathrm{W} \mathrm{W} \mathrm{W}}}/\varLambda ^2 < 2.0\,\text {Te}\text {V} ^{-2}$.

The additional hadronic activity, as well as the efficiencies for gap activity vetos, are studied in a signal-enriched region. Generally reasonable agreement is found between the data and the quantum chromodynamics predictions with the herwig++ parton shower and hadronization model, while the pythia model predictions typically show greater activity in the rapidity gap between the two tagging jets.

## Figures and Tables

**Fig. 20:**
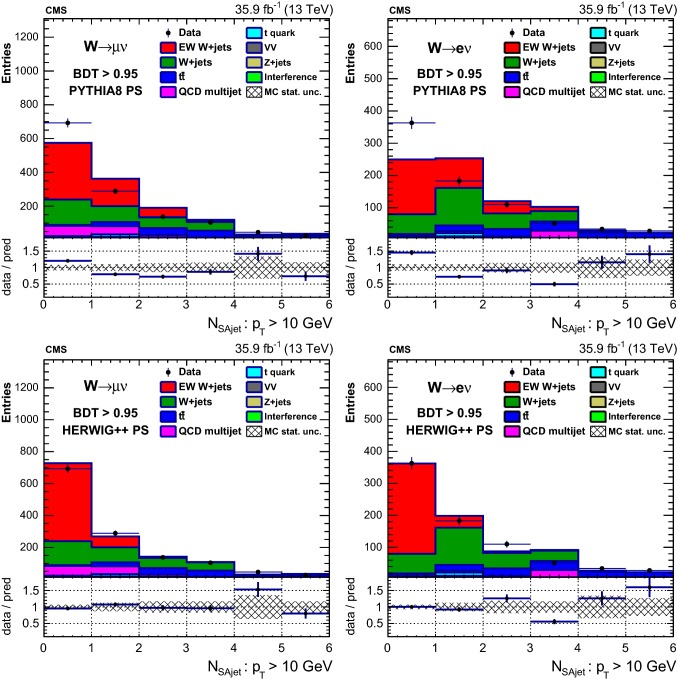
Number of soft activity jets with $p_{\mathrm {T}} > 10\,\text {Ge}\text {V}$ in the rapidity gap for $\mathrm {BDT} > 0.95$ in the muon (left) and electron (right) channels including signal with pythia parton showering (upper) and herwig++ parton showering (lower). In all plots the last bin contains overflow events

**Fig. 21:**
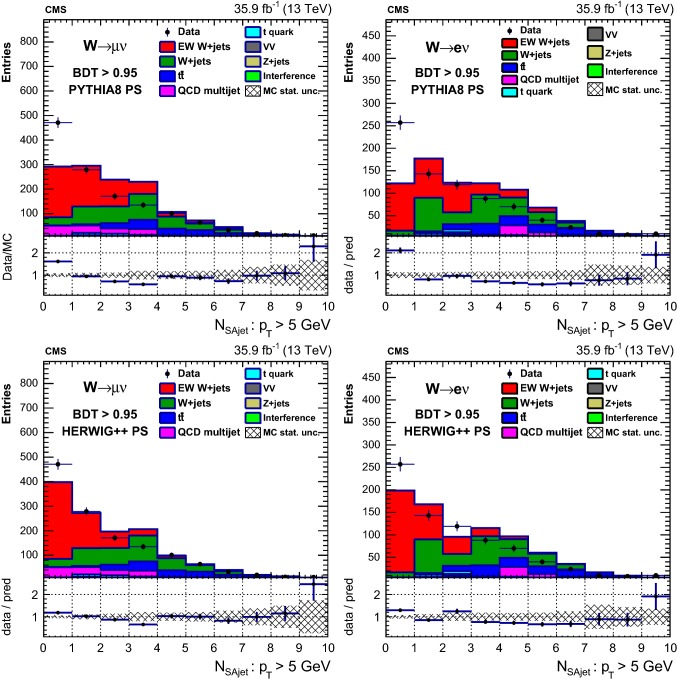
Number of soft activity jets with $p_{\mathrm {T}} >5\,\text {Ge}\text {V}$ in the rapidity gap for $\mathrm {BDT} > 0.95$ in the muon (left) and electron (right) channels including signal with pythia parton showering (upper) and herwig++ parton showering (lower)

**Fig. 22:**
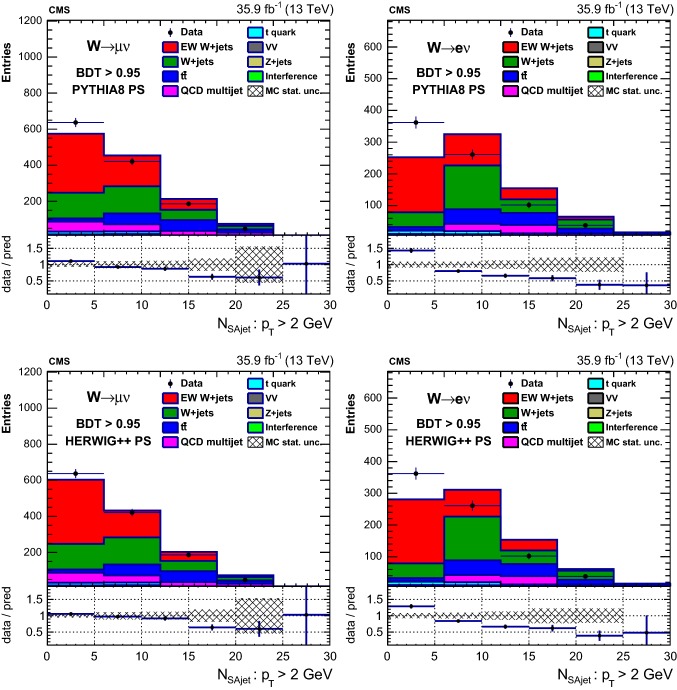
Number of soft activity jets with $p_{\mathrm {T}} >2\,\text {Ge}\text {V}$ in the rapidity gap for $\mathrm {BDT} > 0.95$ in the muon (left) and electron (right) channels including signal with pythia parton showering (upper) and herwig++ parton showering (lower)

**Fig. 23:**
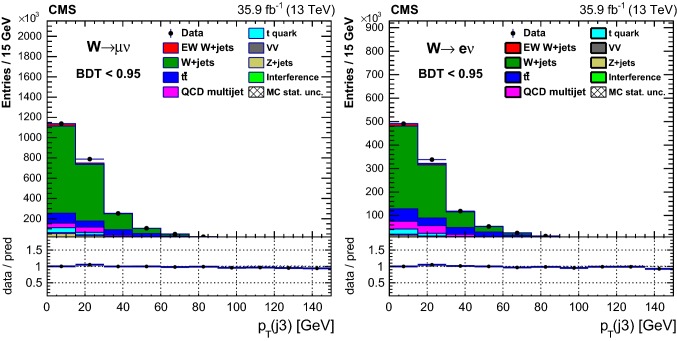
Leading additional jet $p_{\mathrm {T}}$ ($p_{\mathrm {T}}$ (j3)) for BDT $< 0.95$ in the muon (left) and electron (right) channels. In all plots the first bin contains events where no additional jet with $p_{\mathrm {T}} >15\,\text {Ge}\text {V}$ is present

**Fig. 24:**
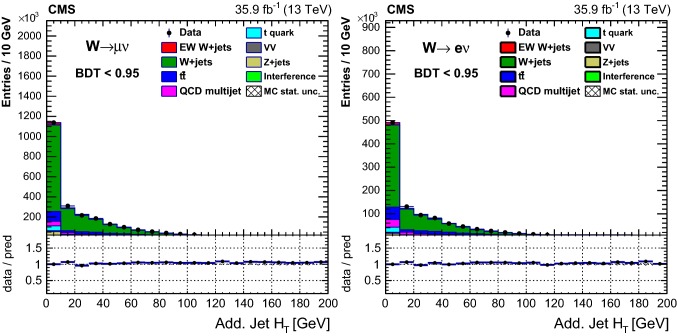
Total $H_{\mathrm {T}}$ of the additional jets for BDT $< 0.95$ in the muon (left) and electron (right) channels. In all plots the fist bin contains events where no additional jet with $p_{\mathrm {T}} >15\,\text {Ge}\text {V}$ is present

**Fig. 25:**
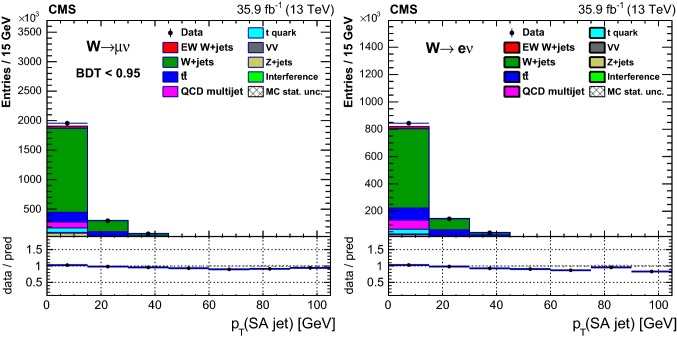
Leading additional soft-activity (SA) jet $p_{\mathrm {T}}$ for BDT $< 0.95$ in the muon (left) and electron (right)

**Fig. 26:**
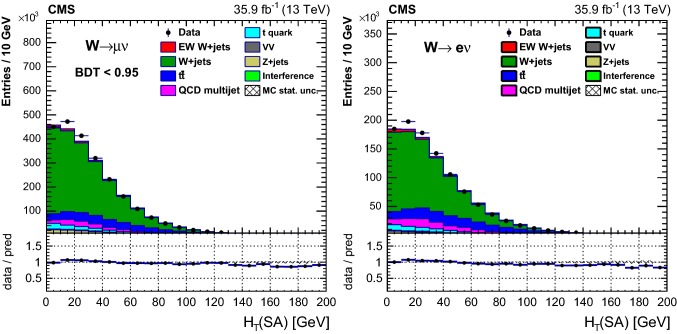
Total soft activity (SA) jet $H_{\mathrm {T}}$ for BDT $< 0.95$ in the muon (left) and electron (right) channels

**Fig. 27:**
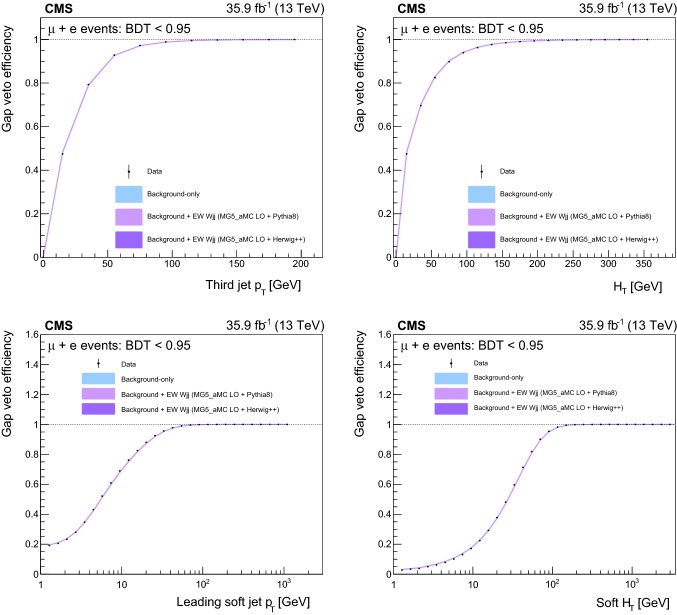
Hadronic activity veto efficiencies in the signal-depleted BDT $< 0.95$ region for the muon and electron channels combined, as a function of the leading additional jet $p_{\mathrm {T}}$ (upper left), additional jet $H_{\mathrm {T}}$ (upper right), leading soft-activity jet $p_{\mathrm {T}}$ (lower left), and soft-activity jet $H_{\mathrm {T}}$ (lower right). The data are compared with the background-only prediction as well as background+signal with pythia parton showering and background+signal with herwig++ parton showering. The uncertainty bands include only the statistical uncertainty in the prediction from simulation. There is very little difference between the predictions due to the small fraction of signal in this region

## Data Availability

This manuscript has no associated data or the data will not be deposited. [Authors’ comment: Release and preservation of data used by the CMS Collaboration as the basis for publications is guided by the CMS policy as written in its document “CMS data preservation, re-use and open access policy” (https://cms-docdb.cern.ch/cgi-bin/PublicDocDB/RetrieveFile?docid=6032&filename=CMSDataPolicyV1.2.pdf&version=2).]

## Additional rapidity gap observables

A set of rapidity gap observables in the high signal purity region $\mathrm {BDT} > 0.95$ is studied in addition to the results described in Section 12. The number of soft activity jets, defined in Section 12.2, in the rapidity gap between the two tag jets is shown for soft activity jet $p_{\mathrm {T}} > 10$, 5, and 2$\,\text {Ge}\text {V}$ in Figures 20, 21, and 22, respectively. These distributions are consistent with the general underestimation of the simulation with respect to data at low activity values, particularly for the pythia parton showering.

## Jet activity in signal-depleted region

Section 12 shows a comparison of the data with simulation with pythia and herwig++ parton showering separately in a high purity signal region with $\mathrm {BDT} > 0.95.$ The agreement of the simulation with data for the background prediction is validated for the rapidity gap observables in the signal-depleted region BDT $< 0.95$, where the signal purity is less than 2%. Figures 23, 24, 25, and 26 show the leading additional jet $p_{\mathrm {T}}$, the total $H_{\mathrm {T}}$ of the additional jets, the leading soft activity jet $p_{\mathrm {T}}$, and the total soft activity jet $H_{\mathrm {T}}$, respectively, in the region BDT $< 0.95$. Good agreement is observed between the background prediction and the data for all observables.

### Hadronic activity vetoes

The efficiency of a hadronic activity veto, as described in Section 12.3, is studied in the signal-depleted $\mathrm {BDT} < 0.95$ region. Figure 27 shows the gap activity veto efficiency of combined muon and electron events in the signal-depleted region when placing an upper threshold on the $p_{\mathrm {T}}$ of the additional third jet, on the $H_{\mathrm {T}}$ of all additional jets, on the leading soft-activity jet $p_{\mathrm {T}}$, or on the soft-activity $H_{\mathrm {T}}$. There is very little difference between the background-only prediction and the predictions including signal with either pythia or herwig++ parton showering due to the very small fraction of signal in this region. Good agreement is observed between the data and the simulation, giving further confidence in the modelling of the background observables for the rapidity gap studies.
